# P38 MAPK activated ADAM17 mediates ACE2 shedding and promotes cardiac remodeling and heart failure after myocardial infarction

**DOI:** 10.1186/s12964-023-01087-3

**Published:** 2023-04-12

**Authors:** Qi Chen, Yilan Li, Bike Bie, Bin Zhao, Yanxiu Zhang, Shaohong Fang, Shuijie Li, Yao Zhang

**Affiliations:** 1 Department of Cardiology, The Second Affiliated Hospital of Harbin Medical University, No. 246 Xuefu Road, Nangang District, Harbin, 150001 Heilongjiang Province China; 2 The Key Laboratory of Myocardial Ischemia, Harbin Medical University, Ministry of Education, No. 246 Xuefu Road, Nangang District, Harbin, 150001 Heilongjiang Province China; 3 Harbin Medical University, No. 157 JianBao Road, Nangang District, Harbin, 150001 Heilongjiang Province China; 4 Department of Biopharmaceutical Sciences, College of Pharmacy, Harbin Medical University, Harbin, 150001 Heilongjiang Province China

**Keywords:** ADAM17, Myocardial infarction, Heart failure, Cardiac remodeling, ACE2

## Abstract

**Background:**

Heart failure (HF) after myocardial infarction (MI) is a prevalent disease with a poor prognosis. Relieving pathological cardiac remodeling and preserving cardiac function is a critical link in the treatment of post-MI HF. Thus, more new therapeutic targets are urgently needed. The expression of ADAM17 is increased in patients with acute MI, but its functional role in post-MI HF remains unclear.

**Methods:**

To address this question, we examined the effects of ADAM17 on the severity and prognosis of HF within 1 year of MI in 152 MI patients with or without HF. In mechanistic studies, the effects of ADAM17 on ventricular remodeling and systolic function were extensively assessed at the tissue and cellular levels by establishing animal model of post-MI HF and in vitro hypoxic cell model.

**Results:**

High levels of ADAM17 predicted a higher incidence of post-MI HF, poorer cardiac function and higher mortality. Animal studies demonstrated that ADAM17 promoted the occurrence of post-MI HF, as indicated by increased infarct size, cardiomyocyte hypertrophy, myocardial interstitial collagen deposition and cardiac failure. ADAM17 knock down significantly improved pathological cardiac remodeling and cardiac function in mice with MI. Mechanistically, activated ADAM17 inhibited the cardioprotective effects of ACE2 by promoting hydrolytic shedding of the transmembrane protein ACE2 in cardiomyocytes, which subsequently mediated the occurrence of cardiac remodeling and the progression of heart failure. Moreover, the activation of ADAM17 in hypoxic cardiomyocytes was dependent on p38 MAPK phosphorylation at threonine 735.

**Conclusions:**

These data highlight a novel and important mechanism for ADAM17 to cause post-MI HF, which will hopefully be a new potential target for early prediction or intervention of post-MI HF.

**Video abstract**

**Supplementary Information:**

The online version contains supplementary material available at 10.1186/s12964-023-01087-3.

## Introduction

Heart failure is the terminal stage of all cardiovascular diseases, which becomes a heavy disease burden worldwide with its high mortality and rehospitalization rates. Post-Myocardial Infarction Heart Failure (post-MI HF) is a common cause of chronic heart failure [1]. Long term hypoxia of cardiomyocytes after MI leads to persistent structural and functional changes, mainly characterized by pathologic cardiomyocyte hypertrophy, myocyte apoptosis, myofibroblast proliferation, and interstitial fibrosis [2]. These cellular changes are the basis for adverse cardiac remodeling, which increases the occurrence of symptomatic chronic HF and even mortality [3]. Systolic function preservation and protection against harmful cardiac remodeling alterations are essential strategies to slow the progression of heart failure after MI. Despite the widespread clinical use of existing ventricular remodeling drugs, the residual risk of cardiac remodeling and heart failure after myocardial infarction is still very high [4]. Novel therapeutic strategies to preserve LV contractile function and limit adverse cardiac remodeling are therefore urgently required to treat patients with HF complicating MI and to improve survival and quality of life.

Post-translational modifications control the biological activities of different target proteins. Proteolytic cleavage of transmembrane proteins is a critical post-translational modification that plays a role in regulating the biological functions of a variety of transmembrane proteins [5–7]. Among these, the metalloproteinase a disintegrin and metalloprotease 17 (ADAM17) has gained a lot of attention in recent years due to its important proteolytic activity involved in regulating inflammation, immunity and fibrosis [8–12].

ADAM17 is known as a metalloproteinase, which can mediate the hydrolytic cleavage of transmembrane proteins and trigger the shedding of the extracellular domain of proteins by activate intracellular kinases such as plk2, MAPK, and PKC [13–15]. More than 10% of cell surface proteins and the majority of transmembrane proteins require proteolytic digestion to release a soluble form for activity [16]. Proteolytic cleavage modulates cellular signals and affects cellular homeostasis depending on the pathway in which the substrates or receptors are involved. As ADAM17 can cleave and thereby activate cytokines and cytokine receptors, such as TNF and IL-6, most studies to date have focused on the role of ADAM17 in inflammation and cancer [17, 18]. In contrast, there is limited information on the cardiovascular aspect of ADAM17, especially in post-MI HF. Studies have demonstrated that ADAM17 is increased in PBMCs of patients with advanced congestive heart failure leads to maturation of TNF-α protein in monocytes, which in turn aggravates the systemic inflammatory response and deterioration of cardiac function in patients [19]. ADAM17 was also found to be essential for post-infarction repair by regulating angiogenesis within 1 week [20]. Whether ADAM17 is only a marker of long-term prognosis of myocardial injury or whether it plays a key role in myocardial remodeling after MI is still unclear.

ACE2 is a key negative regulator of the renin–angiotensin system (RAS) and promotes the degradation of Ang II into the vasodilator, anti-hypertrophic, and anti-inflammatory heptapeptide [21]. Loss of ACE2 in HF and dilated cardiomyopathy greatly aggravates myocardial injury and ventricular remodeling. Conversely, gain-of function experiments with recombinant ACE2, overexpression of ACE2, and Ang 1–7 supplementation have shown protective effects in multiple models of cardiovascular disease, including hypertension, diabetes, and heart failure [22]. The development and deterioration of pathophysiological processes such inflammation, fibrosis, and oxidative stress in myocardial infarction, hypertension, and hypertrophic cardiomyopathy have been demonstrated to be inhibited by ACE2 [22–24]. As a type I transmembrane protein, ACE2 can be hydrolyzed and cleaved by protease [25]. The shedding of ACE2 on the membrane may lead to the loss of ACE2 expression in cardiomyocytes and inhibit the myocardial protection of ACE2. It has been found that ADAM17 could facilitate the cleavage and abscission of soluble ACE2 from cell membranes, limiting ACE2 activity, and exacerbating kidney and lung fibrosis [26, 27]. However, the interaction between ADAM17 and ACE2 in hypoxic cardiomyocytes and the underlying mechanism need to be further clarified.

The p38 family of stress-activated MAPKs has an important role in cardiac signaling and is activated in cardiac pathologies including myocardial infarction, cardiac remodeling, contractile dysfunction, arrhythmia, and heart failure. The previous study demonstrated that p38 MAPK inhibitor suppressed TGF-β-induced SMAD2/3 phosphorylation and SMA expression, and protected the heart from hypertension-induced cardiac remodeling [28, 29]. However, the mechanism of how p38 MAPK regulates ADAM17 in post-MI HF remains unclear.

Herein, we assessed whether the ADAM17 activation is associated with the development of HF in patients with MI. Our studies show that inhibition of ADAM17 expression reduced the shedding of ACE2, which in turn protected against adverse cardiac remodeling and the progression of heart failure. As a result, ADAM17 reactivation after MI plays a significant role in adverse cardiac remodeling and represents a novel therapeutic target with the potential to limit progression to HF among patients with MI.

## Methods

### Clinical study

This prospective observational study included 152 patients with first-time myocardial infarction and no previous heart failure at the Second Affiliated Hospital of Harbin Medical University in 2019. Patients were followed up for 1 year with a median follow-up of 341 days. ADAM17 expression was measured in plasma collected within 24 h after the coronary event. The diagnosis of myocardial infarction was based on the fourth universal definition of myocardial infarction (2018) [30]. The primary endpoint was new-onset HF at 1 year of follow-up. Secondary assessments included a correlation of baseline marker concentrations with echocardiographic measures of systolic dysfunction. New-onset HF was defined as LVEF less than 40% or HF-related hospitalization or HF outpatient diagnosis during follow-up. Exclusion Criteria: (1) Pregnancy; (2) History of heart failure; (3) Severe liver dysfunction, renal dysfunction. Baseline information was collected for all subjects (Additional file 1: Table S1). The trial protocol was approved by the Ethics of Animal Experiments of the Second Affiliated Hospital of Harbin Medical University and was conducted done in accordance with the Declaration of Helsinki (KY2017-249). All patients provided written informed consent.

### ELISA

Serum was separated within 60 min of sample collection and stored at -80℃ until ELISA testing. Serum ADAM17 expression was detected by ELISA according to the manufacturer’s instructions (Human ADAM17 ELISA kit, Human pADAM17 Thr^735^ ELISA kit, Human ACE2 ELISA kit, Mouse ACE2 ELISA kit, Mouse cTnI ELISA kit, JingKang Bio, China).

### Animals

Male C57BL/6 J mice (10–12 weeks old, 25–30 g) were subjected to thoracotomy and left anterior descending coronary artery ligation (LAD ligation, permanent/temporary) using standard approaches. Briefly, the mice were anesthetized with 1–2% isoflurane, left thoracic fourth intercostal space was exposed, and the heart was immediately extruded. The left anterior descending coronary artery was tied below the tip of the left auricle tip with a 7.0 silk thread. TAPI1 was diluted in 5% dimethyllsulfoxide (DMSO) in saline. Mice were orally treated with TAPI-1(10 mg/kg) diluted in DMSO daily for 5 days after MI induction. After 4 weeks, additional echocardiography was performed to assess cardiac function. We confirmed the success of LAD ligation mainly by the following three points. Blood was collected preoperatively and 12 h postoperatively and ELISA was performed to detect troponin I expression in the serum. The expression of cardiac troponin I in myocardial infarction mice was increased (Additional file 1: Table S3). Intraoperatively, the heart ligation area was observed, and pale areas were evident in the ligation area of successfully infarcted hearts. Moreover, 1 month after myocardial infarction, Masson staining observed a large amount of blue collagen deposition in the model group, showing disorganized myocardial fiber arrangement and partial myocardial fiber rupture. All mice were sacrificed with pentobarbital (120 mg/kg body weight, i.p.) at different time points for subsequent experiments. All animal care and handling procedures conformed to the guidelines from Directive 2010/63/EU of the European Parliament on the protection of animals used for scientific purposes. All studies were conducted in accordance with the scheme approved by the Ethics of Animal Experiments of the Second Affiliated Hospital of Harbin Medical University (sydwgzr2020-014).

### Echocardiographic analysis

Mice were anesthetized with 1–2% isoflurane for echocardiographic analysis. The cardiac function of all mice was assessed by transthoracic echocardiograph using a Hitachi Vision 900 system (Hitachi Medical System, Tokyo, Japan) with a 13-MHz transducer. Left ventricular end diastolic diameter and end systolic diameter from M-mode were recorded, and left ventricular ejection fraction (LVEF) was calculated.

### Masson trichrome staining

The animals were sacrificed at 4 weeks after MI, and hearts were fixed with 4% paraformaldehyde overnight and embedded in paraffin. Subsequently, the hearts were sectioned coronally to a thickness of 5 μm. According to the manufacturer’s instructions, fibrosis was detected by Masson's Trichrome Stain Kit (Solarbio, China). Fibrotic areas are stained blue and normal tissue is stained red. The area of fibrosis was calculated as the ratio of the total area of fibrosis to the total area of the section. Fibrosis and infarct size were measured by ImageJ software and determined as a percentage of the total left ventricular area.

### Immunofluorescence staining

Frozen sections of heart tissues were prepared by fixing with OCT (Sakura, USA). Slides were permeabilized by 0.3% Triton for 10 min, blocked by goat serum, and then incubated with anti-ADAM17, cTNT (Abcam, USA) primary antibodies at 4 °C overnight. After rinsing with PBS, fluorescent secondary antibody was added and incubated for 2 h at RT. Nuclei were stained with 4′,6-diamidino-2-phenylindole (DAPI, Beyotime, China) for 5 min. Pictures of samples were taken with a fluorescence microscope.

### Wheat germ agglutinin (WGA) Staining

Five-micrometer paraffin sections of heart samples obtained from mice was used to assess cardiomyocyte hypertrophy. Antigen retrieval was performed on paraffin sections using sodium citrate antigen retrieval solution. Sections were stained with 50 μg/ml WGA (Sigma, USA) to measure cardiomyocyte cross-sectional area. After transfection and hydrogen peroxide stimulation, H9C2 cells were fixed with 4% paraformaldehyde and cells were incubated with 50 μg/ml WGA for 30 min at 37 °C. Images were observed by fluorescence microscopy. Surface area was measured with ImageJ and the value was expressed as fold change relative to control.

### Cell culture and treatment

H9C2 cardiomyocytes were cultured in DMEM medium (Sigam, USA) containing 10% fetal bovine serum (BD, America) and 100 U/ml penicillin and 100 μg/ml streptomycin (Beyotime, China). Cells were treated with hydrogen peroxide at indicated. Isolation and culture of neonatal rat primary cardiomyocytes was performed as described previously [31]. Briefly, heart tissues of neonatal rats were dissociated with trypsin and cells were plated for 1 h into tissue culture dishes in DMEM with 10% FBS to reduce the number of nonmyocytes. Cells that did not attach to the plates were aspirated and plated into 6-well culture plates. Primary cardiomyocytes were cultured in the same growth medium as H9C2 cells. ADAM17 knockout in cardiomyocytes was achieved by transfection of siRNA (sequence: ADAM17 siRNA AUCAAGGCUAAAUUGCUCCTT, Negative control UUCUCCGAACGUGUCAC GUTT) using Lipofectamine 3000 (Invitrogen, USA) according to the manufacturer’s instructions. Cells were subsequently processed 24 h after transfection.

### Annexin V-FITC/PI double-staining

Flow cytometry was used to assess cell apoptosis with an Annexin V-FITC/PI staining kit (BD, USA) following the manufacturer’s instructions. Briefly, take 5 × 10^5–6^ cells/ml and add PBS to suspend the cells. Centrifuge at 1000 rpm, 4 °C for 10 min, discard the supernatant, and repeat 3 times. Cells were resuspended in 200 μl binding buffer, 10ul Annexin V-FITC was added and mixed gently. After 15 min of reaction in the dark at room temperature, 5 μl of propidium iodide was incubated for 5 min and detected within 1 h.

### CCK8 assays

After transfection and hydrogen peroxide stimulation, 10 μl Cell Counting Kit 8 (CCK8, Dojindo, Japan) was added to each well for 2 h. Cell viability was measured at absorbance at 450 nm with a microplate reader (Bio-Rad Laboratories, Benicia, CA, USA).

### Concentration of cell supernatants

The treated cell supernatant was collected. These supernatants (300–400 ml/well) were concentrated using Amicon Ultra Centrifugal Filters Ultra-4 (EMD Millipore, USA) according to the manufacturer's specifications. The concentrated supernatant was mixed with loading buffer and subjected to western blotting.

### Western blot

Cells or heart tissue were lysed using RIPA lysis buffer (Beyotime, China). Protein concentration was quantified using a BCA protein-assay kit (Beyotime, China). Protein (30 μg) was separated with 10% SDS-PAGE and then transferred into PVDF membranes (GE Amersham, America). After blocking with 5% skim milk, the membranes were incubated with primary antibodies (anti-ADAM17 1: 1000; anti-phospho-ADAM17 (pThr735), Abcam, USA. anti-collagen I 1: 500, anti- collagenIII 1: 500; anti-TGF-β 1: 1,000; P38 1: 500; pP38 1: 500; ERK1/2 1: 500 p-ERK1/2 1: 300, Wanleibio, China) at 4 °C overnight. Then, membranes were incubated with secondary antibodies at room temperature for 1 h. Protein bands were visualized using BeyoECL Plus (Beyotime, China) and measured using IPP Image-Pro Plus software.

### Co-immunoprecipitation

Co-immunoprecipitation (co-IP) was performed as described [32]. Cells were collected and lysed at 4 °C with IP buffer (Beyotime, China). Clarified extracts were then mixed with 2 μl of specific antibody and rotated for 2 h at 4 °C. Following this, 50 μl of Protein A/G Immunomagnetic beads (Bimake, China) equilibrated in IP buffer were added to the extracts and further rotated for 1 h at 4 °C. Beads were washed twice with IP buffer and finally resuspended in SDS-sample buffer. Software Antibodies against ADAM17 (#3976, Cell Signaling Technology, 1:100) and ACE2 (#AF933, R&D, 1:100) were used.

### Real-time quantitative PCR (RT-qPCR)

Total RNA from myocardial tissue and cultured cells was extracted by Trizol (Thermo, USA). RNA was reverse transcribed into cDNA with reference to reverse transcription kit (Roche, Basel, Switzerland). PCR was conducted using Bestar Sybr Green qPCR Master Mix (DBI Bioscience, Germany) at the following settings: 40 cycles of 10 s at 95 °C, 30 s at 60 °C, and 30 s at 72 °C. Results were normalized to β-actin expression. The primer sequences used for RT-qPCR are listed in Additional file 1: Table S2.

### ACE2 activity assay

Collect H9C2 cells or tissues by centrifugation, add 100–200 μl Lysis Buffer per 1 million cells, fully lyse cells on ice for 5–10 min. The supernatant was collected by centrifugation, homogenizing and adding 100 μl of Lysis Buffer per 10 mg of sample. Centrifuge at ~ 12,000×*g* for 3–5 min at 4 °C and save the supernatant [25]. The activity of ACE2 in the samples was detected by ACE2 activity fluorescence detection kit (Beyotime, China).

### Protein and RNA docking program

To evaluate the predicted interaction between ADAM17 and ACE2, we first download the protein structure of ADAM17(PDB ID: 2FV9) and ACE2(PDB ID: 6M17) from PDB database (www.rcsb.org). Zdock software was used for proteins docking, and select the conformation with the highest docking score. The docking model is shown in Additional file 1: Fig. S3.

### Statistical analysis

For the clinical study, continuous variables were expressed as mean ± standard deviation or median [IQR], and Student's t test or Mann–Whitney U was used for comparison between groups. Categorical variables were expressed as frequency and percentage (%), and Chi-square test was used for comparison between groups. Receiver operating characteristic (ROC) curve analysis was carried out based on gene expression levels. The area under the ROC curve (AUC) was calculated to assess the specificity and sensitivity of single genes and their combination via the binary logistic regression analysis. The correlation between ADAM17 and EF% was analysed with the Spearman rank test. Cox proportional hazard models were used to identify the association of ADAM17 with the risk of all-cause death. 4 models were adjusted for potential confounders and independent predictors of death. All data in the figures are presented as means ± SEMs unless otherwise specified. The data were analyzed using GraphPad Prism-8 statistic software. Variances between two groups were analyzed by two-sample *t*-tests. For other comparisons, analysis of variance was calculated by one-way ANOVA. Differences with *P* values of less than 0.05 were considered statistically significant.

## Results

### High ADAM17 in MI patients was associated with left ventricular dysfunction and heart failure

**Fig. 1:**
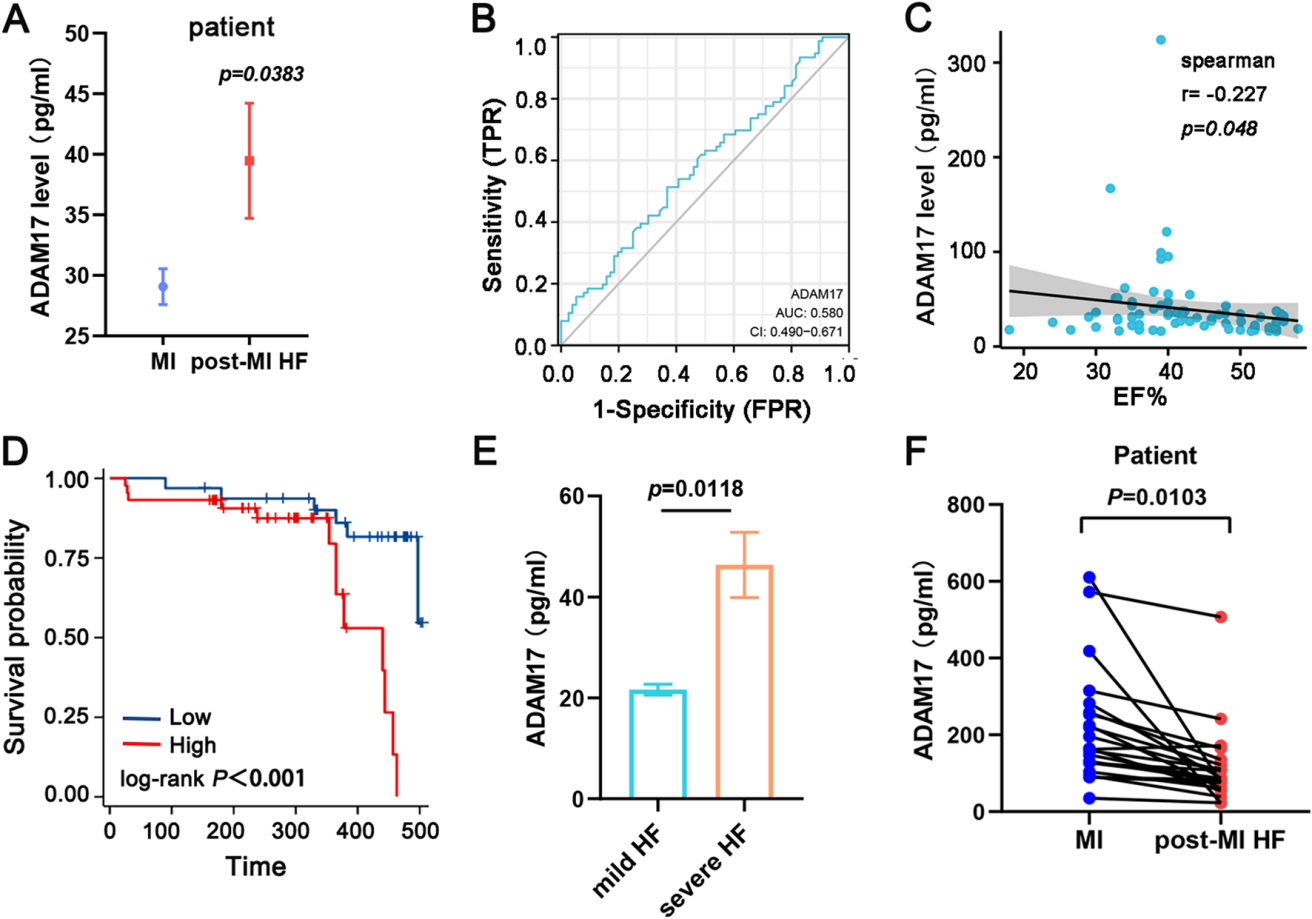
High expression of ADAM17 is associated with the occurrence of post-MI HF*.* **A** The expression of ADAM17 in peripheral blood of patients was detected by ELISA. **B** Spearman correlation between ADAM17 expression of peripheral blood and EF% in patients with MI. **C** Receiver-operating characteristic curve (ROC) for ADAM17 and their corresponding area under the curve (AUC) values. **D** The post-MI HF patients were divided in two groups according by ADAM17 expression level. Mortality rates were analysed using the Kaplan–Meier method. **E** Post-MI HF patients was divided into two subgroups by NYHA functional class (I and II vs. III and IV), ADAM17 levels was detected by ELISA in two HF subgroups. **F** Evolution of serum ADAM17 levels after HF during 1-year follow-up. Data shown as mean ± SEM. *P < 0.05; **P < 0.01, ***P < 0.001

A total of 152 patients with onset of new MI and no previous HF at the Second Affiliated Hospital of Harbin Medical University were enrolled in the study with a 1-year follow-up and a median follow-up of 341 days. Within 24 h of the coronary incident, plasma was taken and the expression of ADAM17 was evaluated. All patients completed rapid revascularization and aggressive treatment. In all cases, ischemia-related arteries were revascularized using drug-eluting stents (n = 101) or balloon angioplasty alone (n = 51). Baseline clinical characteristics are shown in Additional file 1: Table S1, which includes baseline characteristics, laboratory covariates, and therapeutic features. Patients with heart failure after MI were older and had a history of hypertension. Higher baseline ADAM17 expression was associated with a greater the risk of having HF within 1 year following MI,* p* = 0.038. (Fig. 1A). In the ROC analysis, the area under the curve (AUC) of ADAM17 (for predicting the occurrence of HF) was 0.58. ADAM17 had a sensitivity of 51.3%, a specificity of 63.2%, a negative predictive value of 56%, a positive predictive value of 58.2%, and a Youden index of 0.145 in predicting HF after 1 year in patients with MI (Fig. 1B). We then analyzed the correlation between ADAM17 expression and left ventricular ejection fraction (EF) values in patients with HF after MI. ADAM17 expression was inversely correlated with EF (Fig. 1C).

Then, we analyzed the correlation of ADAM 17 with prognosis in post-MI HF patients. During the follow-up, 19 all-cause deaths occurred in 76 patients with post-MI HF. The patients were divided in two groups according to high and low ADAM17 expression level, separated by the median value. In a Kaplan–Meier analysis with log-rank test, all-cause mortality was significantly higher in the high ADAM17 group (≥ 27.94 pg/ml, n = 44) than in the low ADAM17 group (< 27.94 pg/ml, n = 32) (Fig. 1D, *P* = 0.0006, HR 0.239; 95% confidence interval (CI), 0.093–0.617). These results were further confirmed in a Cox proportional regression analysis (Table 1). Univariable analysis showed a 5.801 (95% CI 1.936–17.384l) higher risk for all-cause deaths increase in baseline ADAM17. This result remains stable after adjusting for incremental numbers of confounders throughout models 1–4 (model1: HR3.544, 95% CI 1.119–11.223; model2: HR3.582, 95% CI 1.128–11.370; model 3: HR 4.342, 95% CI 1.271–14.834; model 4: HR5.620, 95% CI 1.406–22.470) (Table 1, model 1–4). When the post-MI HF group was divided into two subgroups by NYHA functional class (I and II vs. III and IV), levels of ADAM17 in peripheral blood plasma were significantly higher in severe HF patients (NYHA III or IV) than in mild HF patients (Fig. 1E). These data suggest that high level of ADAM17 correlates with poor prognosis in post-MI HF patients, which were an independent predictor for mortality in post-MI HF patients.

**Table 1:** Cox regression model for the association between serum ADAM17 and all-cause mortality

	ADAM17 (pg/ml)		*P* value
	Low (n = 32)	High (n = 44)	
No. deaths	6	13	
Unadjusted	Ref	5.801 (1.936–17.384)	0.002
Model 1	Ref	3.544 (1.119–11.223)	0.031
Model 2	Ref	3.582 (1.128–11.370)	0.030
Model 3	Ref	4.342 (1.271–14.834)	0.019
Model 4	Ref	5.620 (1.406–22.470)	0.015

Unadjusted model adjusted for none. Model 1 is adjusted on age and gender. Model 2 is adjusted on age, gender, co-morbidities (hypertension and diabetes mellitus). Model 3 is adjusted on age, gender, co-morbidities (hypertension and diabetes mellitus), biological variables (NT-proBNP, cTNI, eGFR, hs-CRP, TC and TG). Model 3includes variables of Model 3, treatment (PCI information, β-blockers, ACEIARB, ARNI, and diuretics)

To further evaluate changes in ADAM17 expression in peripheral blood of patients with post-MI HF. 22 blood samples of post-MI HF patients were collected at the time of diagnosis of heart failure. Mean baseline concentrations of ADAM17 were significantly decreased after diagnosis of HF, suggesting that ADAM17 level may decreased over time in post-MI HF patients (Fig. 1F).

### Myocardial ADAM17 expression is elevated in infarcted heart

**Fig. 2:**
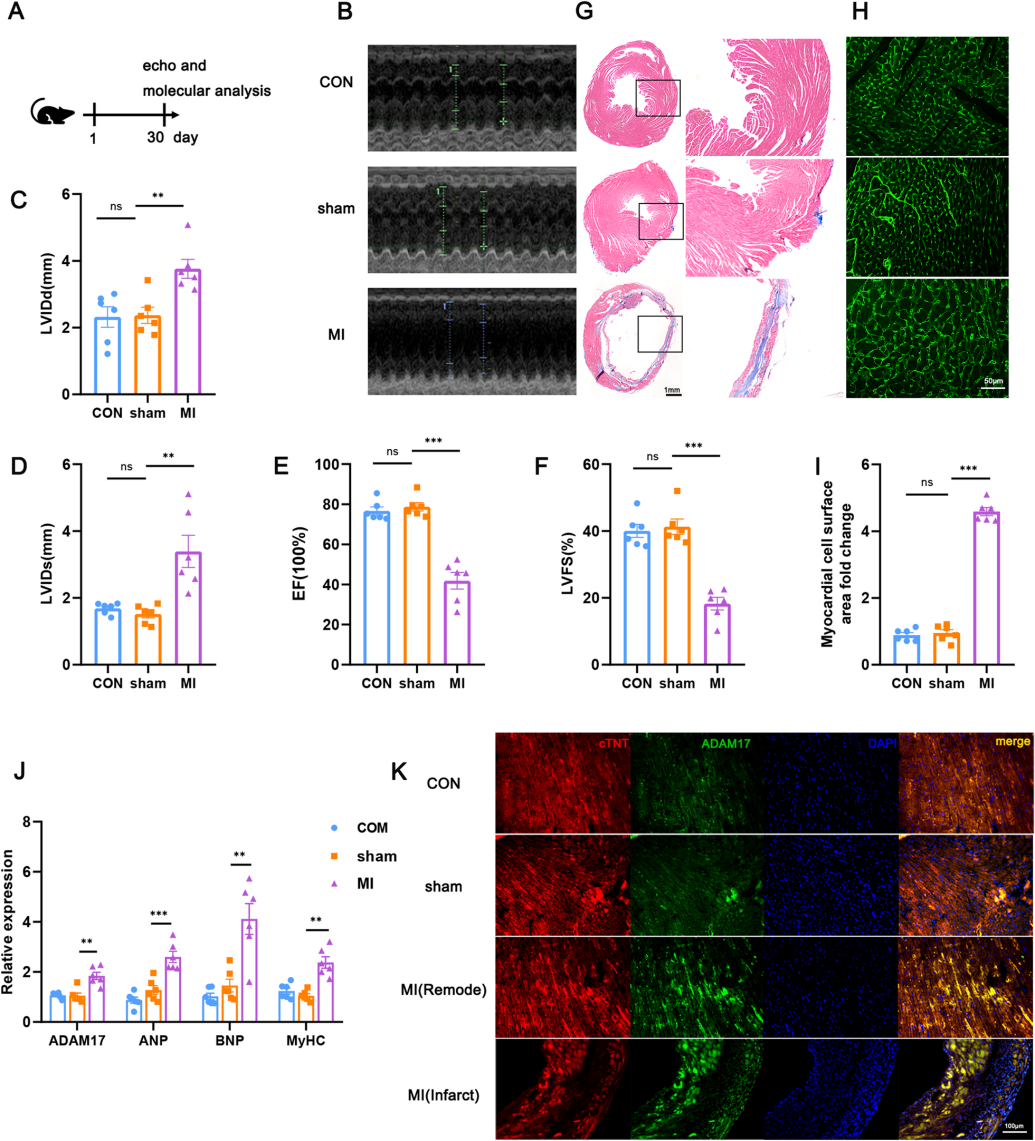
ADAM17 expression was increased in myocardium of post-MI HF mice. **A** WT C57BL/6 mice were subjected to MI surgery and sacrificed after 30 days. **B**–**F** Echocardiographic analysis images of left ventricular (LV) M-mode echocardiograms 30 days after surgery. LV Internal diastolic diameter (LVIDd) (C), LV internal dimension systole (LVIDs) (D), Ejection fraction (EF) (E), and fractional shortening (FS) (F) were measured from different groups. **G** Representative Masson’s trichrome staining of the hearts from different groups. **H**–**I** WGA staining to measure cardiomyocyte surface area and its quantification. **J** mRNA expression levels of ADAM17, ANP, BNP, and MyHC of different groups mice heart was measured by RT-qPCR. **K** Immunofluorescence for cTNT (red) ADAM17 (green) and DAPI (blue) nuclei staining of histological sections in remote and infarct zone of the MI mice heart. CON, WT control; sham, sham operation; MI = MI surgery. N = 6 biological replicates. Data shown as mean ± SEM. Derived by two-sample *t*-test, *P < 0.05; **P < 0.01, ***P < 0.001

Wild-type (WT) C57BL/6 mice were subjected to sham operation or MI and euthanize at 30 days after surgery (Fig. 2A). According to echocardiography, MI mice had more severe cardiac dysfunction than sham mice (Fig. 2B). After 30 days, MI mice had considerably more adverse ventricular chamber dilatation, both in systole and diastole (Fig. 2C, D). The left ventricle fractional shortening (LVFS) and preserved ejection fraction (EF) both showed decreased ventricular performance in the presence of abnormal cardiac enlargement (Fig. 2E, F). The scar was markedly thinned and comprised a much larger percent circumference of LV post-MI (Fig. 2G). WGA staining showed increased pathological hypertrophy of cardiomyocytes in the non-infarcted region (Fig. 2H, I). Elevated HF indicators (ANP, BNP, and MyHC) were also observed at the same time (Fig. 2J). All of these changes suggested that 30 days after MI, the mice developed severe cardiac remodeling and even heart failure. Most strikingly, the expression of ADAM17 mRNA was significantly increased in post-MI HF cardiocytes (Fig. 2J). The ADAM17 protein localization in the myocardium was further detected using immunofluorescence, which revealed that ADAM17 levels were considerably higher in infarct and remote areas, respectively, than in sham controls (Fig. 2K). These finding suggest that ADAM17 may mediate the pathogenesis of HF after MI.

### ADAM17 deficiency reduce cardiac dysfunction and remodeling in post-MI HF mice

**Fig. 3:**
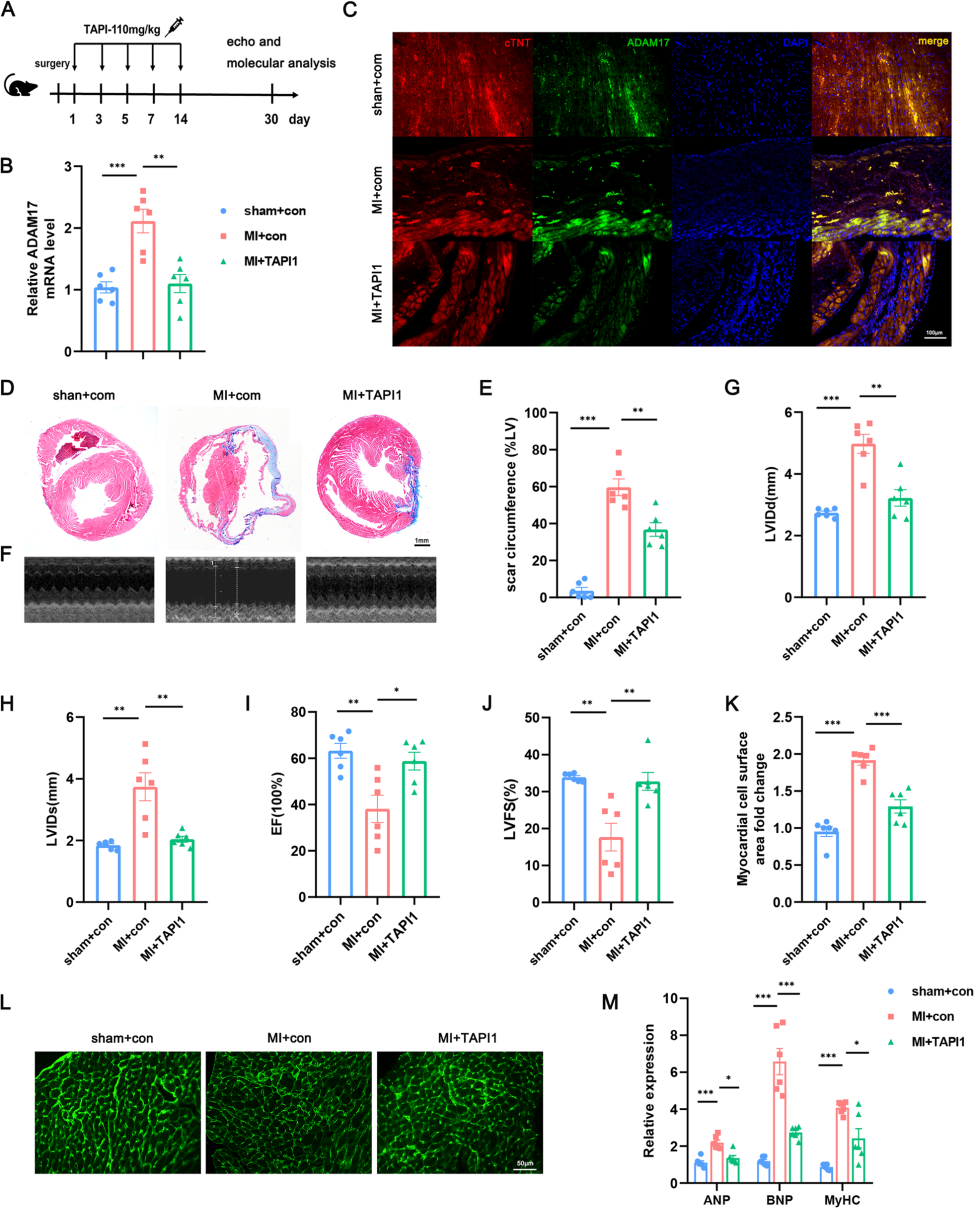
Gene silencing of ADAM17 reduced infarct size and improve cardiac function in MI mice. **A** Schematic diagram of the experimental design. The ADAM17 inhibitor TAPI-1 or a comparable amount of DMSO were gavaged into mice on days 1, 3, 5, 7, and 14. Mice were sacrificed on day 30 after the first treatment. **B** RT-qPCR analysis of the expression of ADAM17 after administration. **C** Immunofluorescence of cTNT (red), ADAM17 (green) and DAPI (blue) in the infarcted area of the MI mice heart. **D**, **E** Masson trichrome staining of heart cross-sections (D). Scar circumference was measured as the percentage over the area of LV myocardium (E). F–J Echocardiographic analysis images on 30 days after treatment (F). Statistical charts for LVIDd(G), LVID(H), EF (I) and FS (J) are shown. **K**–**L** WGA staining to measure cardiomyocyte surface area and its quantification. **M** RT-qPCR analysis of the expression of ANP, BNP, and MyHC of the MI mice Myocardium. N = 6 biological replicates. Data shown as mean ± SEM. Derived by two-sample t-test, *P < 0.05; **P < 0.01, ***P < 0.001

To examine whether ADAM17 has a direct pathogenic role in post-MI HF, we evaluated the effects of ADAM17 blockade on cardiac function in MI mice. Briefly, we induced MI in WT C57BL/6 mice and administered 10 mg/kg of the specific ADAM17 blocker TAPI-1 [33–36] to the mice at 3, 5, 7, and 14 days after the MI. The mice were then euthanized 30 days later (Fig. 3A). Sham-operated mice and MI mice treated with DMSO were used as controls. Loss of ADAM17 in cardiomyocytes was confirmed by RT-qPCR (Fig. 3B). The expression of ADAM17 was also downregulated in the myocardial infarction area, according to immunofluorescence detection (Fig. 3C). Mice treated with ADAM17 inhibition had less interstitial fibrosis and fewer infarction overall compared to the control group (Fig. 3D, E). Consistent with reduced fibrosis, cardiac dysfunction was significantly improved, including increased EF and FS, decreased LVIDd and LVIDs (Fig. 3F–J). This facilitative effect on myocardial remodeling was further confirmed by measuring myocyte size (Fig. 3K, L) and decreasing gene expression of ANP, BNP and the markers of cardiac hypertrophy, MyHC (Fig. 3M). Taken together, ADAM17 deletion decreases infarct size and enhances cardiac function in mice with post-MI HF.

### ADAM17 is upregulated in injured cardiomyocyte

**Fig. 4:**
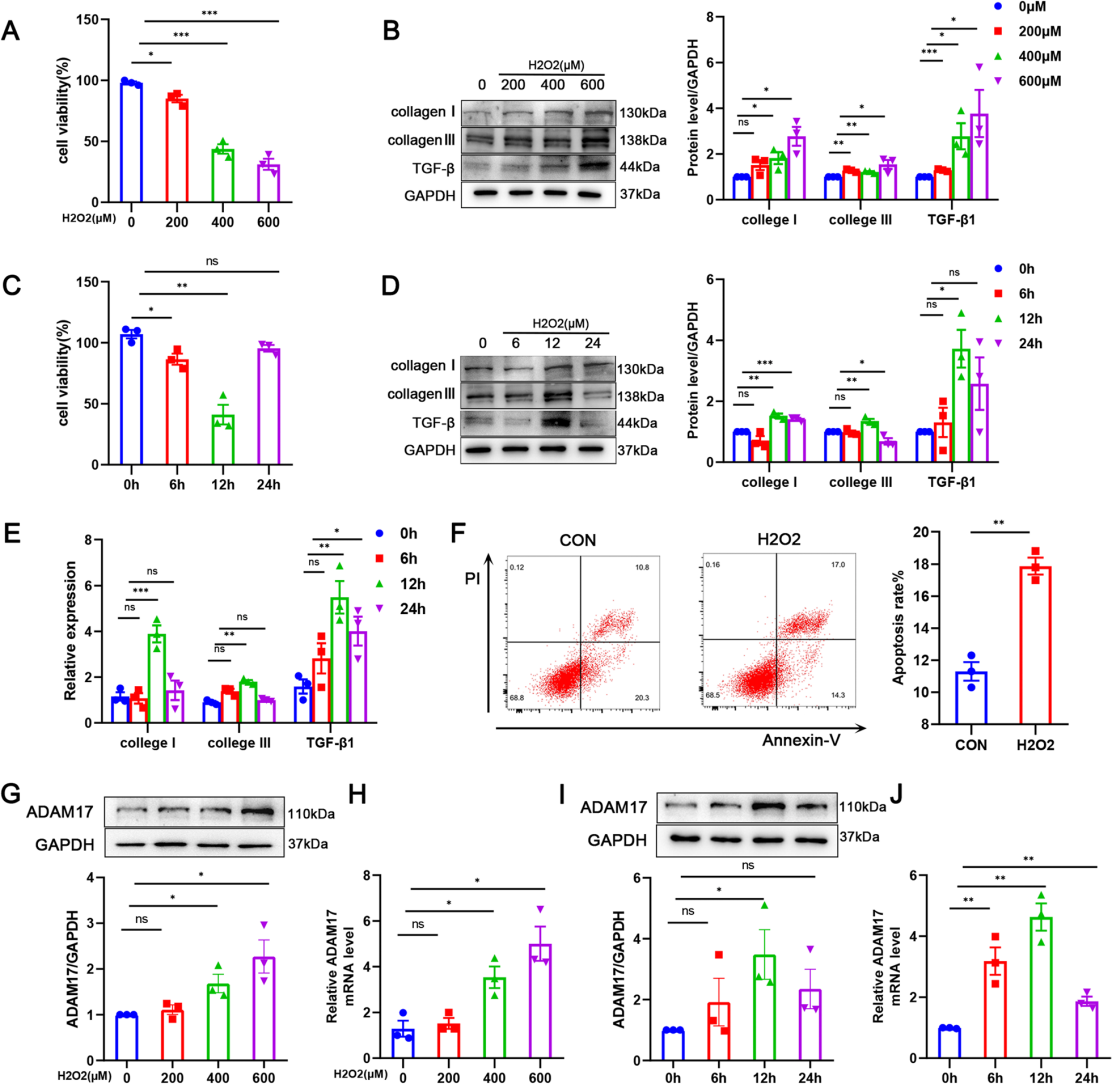
Expression pattern of ADAM17 in hydrogen peroxide-treated cardiomyocytes. **A** CCK8 detects H9C2 cardiomyocyte viability after stimulation with different concentrations. **B** Western blot measurement of ADAM17 and myocardial remodeling-related makers collagenI, collagenIII and TGF-β1 in H9C2 cardiomyocytes treated with different concentrations of hydrogen peroxide. **C** Cell viability of H9C2 cardiomyocytes treated with 600 μM hydrogen peroxide for the indicated time was detected by CCK8. **D** CollagenI, collagenIII and TGF-β1 protein levels of hydrogen peroxide-treated H9C2 cardiomyocytes at the indicated time were detected by western blotting. **E** RT-qPCR analysis of collagenI, collagenIII and TGF-β1 mRNA levels in hydrogen peroxide-treated H9C2 cardiomyocytes. **F** Representative images were taken from the above cells stained by Annexin V/propidium iodide(right). Cell apoptosis (%) was measured by cytometry(left). **G** western blot measurement of ADAM17 level in H9C2 cardiomyocytes treated with different concentrations of hydrogen peroxide for 12 h. **H** RT-qPCR analysis of ADAM17 mRNA level in H9C2 cardiomyocytes treated with different concentrations of hydrogen peroxide for 12 h. **I** western blot measurement of ADAM17 level in H9C2 cardiomyocytes treated with 600 μM hydrogen peroxide for the indicated time. **J** RT-qPCR analysis of ADAM17 mRNA level in H9C2 cardiomyocytes treated with 600 μM hydrogen peroxide for the indicated time. N = 3 independent cell preparations. Data shown as mean ± SEM. Derived by two-sample t-test, *P < 0.05; **P < 0.01, ***P < 0.001

Hydrogen peroxide was used to cause cell death in H9C2 cardiomyocytes in order to study the function and mechanism of ADAM17 in damaged myocardium, which has been utilized to simulate myocardial injury in myocardial infarction as it leads to apoptosis and necrosis of cardiomyocytes [37–39]. After 12 h treatment, cell viability of the H9C2 cardiac cell was significantly decreased in a dose-dependent manner (Fig. 4A). The expression of collagenI, collagenIII and TGF-β1, markers associated with myocardial remodeling, increased progressively (Fig. 4B). Based on the cell viability status of cardiomyocytes at a concentration of 600 μM, we evaluated the effect of hydrogen peroxide on cardiomyocytes after different times treatment. After treatment, the myocardial cell activity was reduced (Fig. 4C) while the protein and mRNA expression levels of collagenI, collagenIII, and TGF-β1 increased (Fig. 4D, E). The cells received their ideal amount of stimulation for 12 h. Thus, we selected 600 μM stimulation for 12 h as the stimulation condition in the subsequent experiments. Consistent with previous results, annexin V/propidium iodide (PI) staining showed an increased cardiomyocyte apoptosis after stimulation (Fig. 4F). We then detected the expression changes of ADAM17 in hydrogen peroxide-treated cardiomyocytes. Similar to the cardiac remodeling index, ADAM17 protein and mRNA level were increased in stimulated myocardium and positively linked with stimulation concentration, according to Western blot and RT-qPCR analysis (Fig. 4G, H). Peak expression was attained after 12 h of optimum stimulation with 600 μΜ hydrogen peroxide (Fig. 4I, J). Similar results were obtained in rat primary cardiomyocytes and primary cardiac fibroblasts (Additional file 1: Figure S1A–D). These results suggest that upregulation of ADAM17 in hydrogen peroxide stimulated cardiomyocyte injury may be involved in regulating cardiomyocyte remodeling.

### ADAM17 deletion reduces cardiomyocyte apoptosis and remodeling

**Fig. 5:**
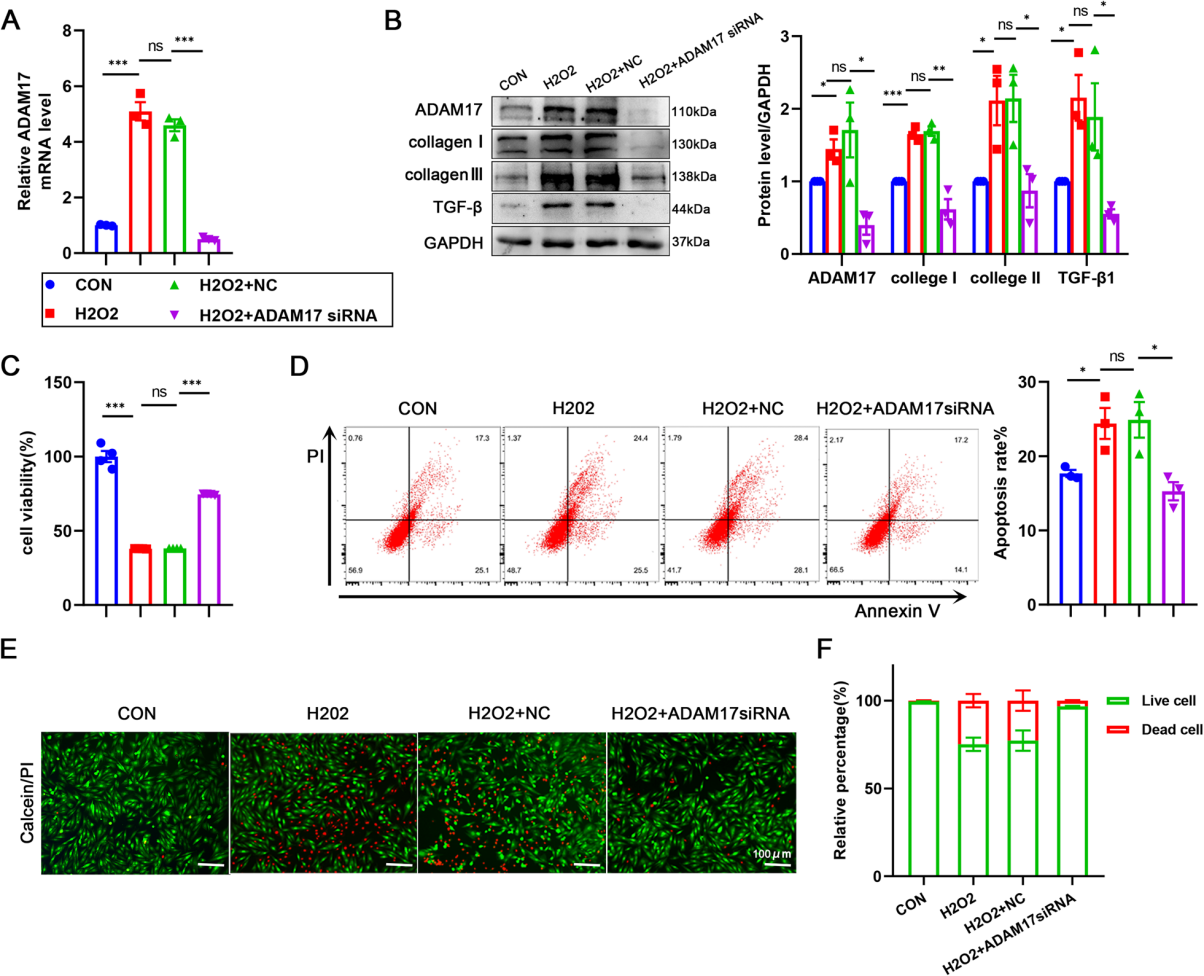
Silencing ADAM17 reduces cardiomyocyte apoptosis and remodeling. H9C2 cardiomyocytes were transfected with ADAM17 siRNA or siRNA control (NC) for 6 h and treated with 600 μM hydrogen peroxide for 12 h. **A** The ADAM17 mRNA expression were detected by RT-qPCR in H9C2 after treatment as above (n = 3). **B** Western blot analysis of ADAM17, collagenI, collagenIII and TGF-β1 protein level in H9C2 cardiomyocytes (n = 3). **C** The cell viability was detected by CCK8 assay (n = 4). **D** Flow cytometric analysis of annexin V/PI double stained cells after hydrogen peroxide treatment (left). Histogram indicating the percentage of later apoptosis cells in 4 groups (right) (n = 3). **E**, **F** Fluorescence microscopic images of cells stained with a calcein-AM/PI double staining kit (E). The live (green) and dead (red) cell percentages quantified from fluorescent images (F) (n = 3). Data shown as mean ± SEM. Derived by two-sample t-test, *P < 0.05; **P < 0.01, ***P < 0.001

We transfected siRNA to inhibit ADAM17 expression in H9C2 cardiomyocytes in order to directly assess ADAM17's role in cardiomyocytes. After transfection with ADAM17 siRNA, a decrease in both ADAM17 mRNA and protein levels was observed, but no change in the siRNA NC group (Additional file 1: Figure S3B, C). To confirm the potential impact of ADAM17 on damaged cardiomyocytes, cardiomyocytes were stimulated with hydrogen peroxide for 12 h after transfection of the ADAM17 siRNA. Then, ADAM17 expression was dramatically downregulated in stimulated cardiomyocytes (Fig. 5A, B). After ADAM17 deletion, western blotting revealed that the expressions of collagen I, collagen III, and TGF-β1 were significantly decreased (Fig. 5B). CCK8 detected an increase in cardiomyocyte viability after ADAM17 knockdown (Fig. 5C). Apoptosis of cells was examined using flow cytometry. Apoptosis was reduced after inhibition of ADAM17 expression, suggesting that inhibition of ADAM17 protected cardiomyocytes from hydrogen peroxide-stimulated apoptosis (Fig. 5D). The protective effect of ADAM17 inhibition on cardiomyocytes was further verified by calcein AM/PI double staining, in which it was determined that live cells stained with calcein AM were green and dead cells stained with PI were red(Fig. 5E). 24.87% of cardiomyocytes died after hydrogen peroxide stimulation. The number of cardiac deaths was considerably decreased after ADAM17 expression was inhibited (Fig. 5F). These results suggest that inhibition of ADAM17 can alleviate hydrogen peroxide-induced cardiomyocyte apoptosis and pathological remodeling.

### ADAM17 mediated shedding of ACE2 in injured cardiomyocyte

**Fig. 6:**
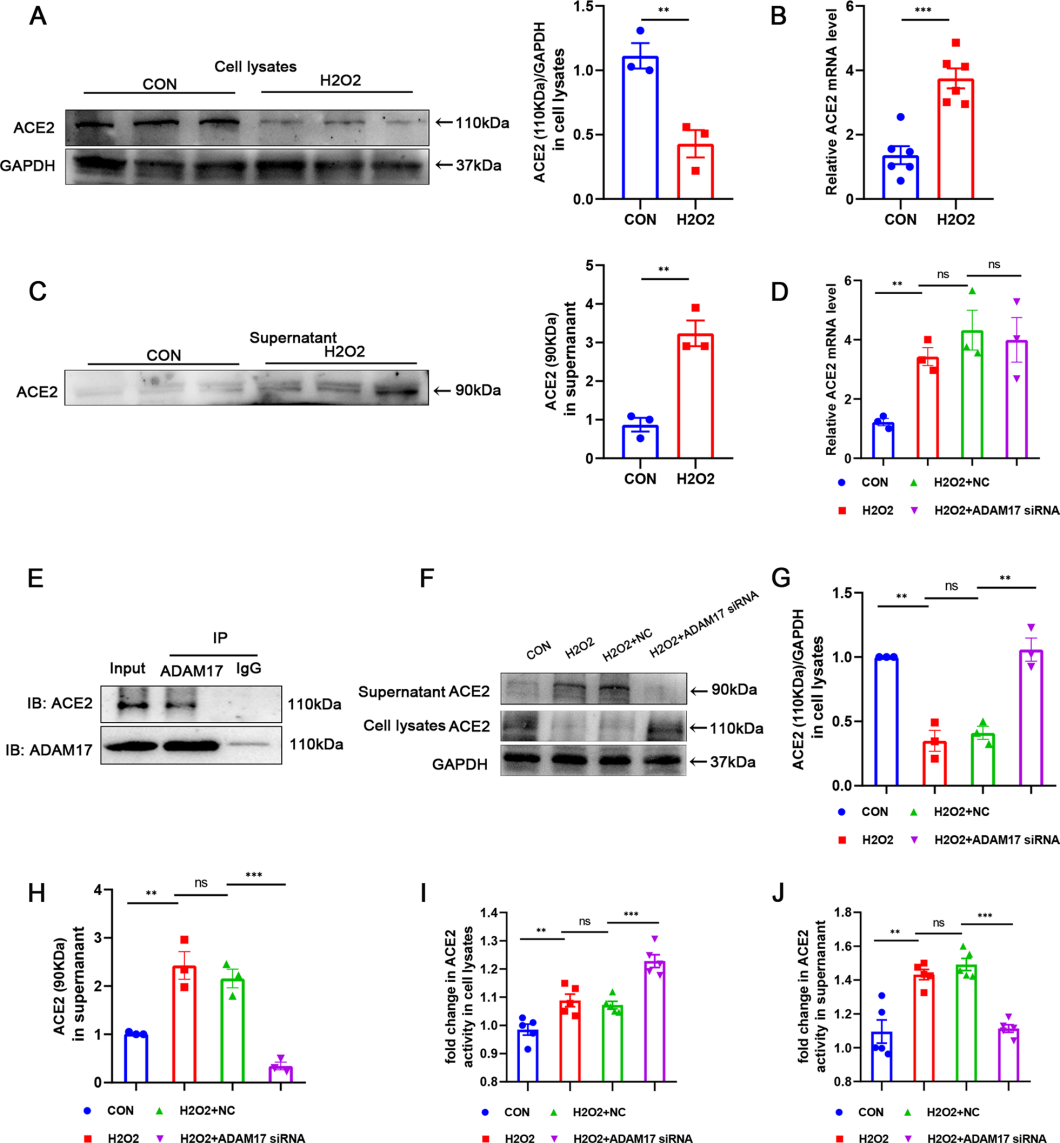
ADAM17 promotes ACE2 shedding in injured cardiomyocyte. **A** Western blot analysis of ACE2 protein level in H9C2 cardiomyocytes treated with 600 μM hydrogen peroxide for 12 h (n = 3). **B** RT-qPCR analysis of ACE2 mRNA level. **C** Western blot analysis of the expression of released ACE2 protein (90 kDa) in the supernatant of cardiomyocytes (n = 5). **D** After transfection of ADAM17 siRNA to inhibit ADAM17 expression, The mRNA expression level of ACE2 was detected by RT-qPCR (n = 3). **E** Co-immunoprecipitation of ADAM17 and ACE2 in H9C2 cardiomyocytes. **F**–**H** The protein level of ACE2 in cardiomyocytes lysate and supernatant was detected by western blotting (n = 3). **I** ACE2 activity in cardiomyocytes lysate was detected by the ACE2 activity fluorometric assay (n = 5). **J** ACE2 activity in cardiomyocyte supernatant was detected by the ACE2 activity fluorometric assay (n = 5). Data shown as mean ± SEM. Derived by two-sample t-test, *P < 0.05; **P < 0.01, ***P < 0.001

Given that ADAM17 has been reported to mediate proteolytic cleavage of ACE2, we next investigated whether ACE2 is involved in the role of ADAM17 in myocardial injury after MI. Protein structure was retrieved from the PDB database and the interaction mode between ACE2 (PDB ID: 6M17) and ADAM17 (PDB ID: 2FV9) was investigated using Zdock (Additional file 1: Figure S3A). The docking results showed a high Zdock docking score for both proteins (1695.903). The residues around the protein protein interaction interface can form a large number of hydrophobic interactions (Additional file 1: Figure S3B) and hydrogen bonds (Additional file 1: Figure S3C). These non-covalent connections suggest that ADAM17 and ACE2 interact to generate stable protein–protein complexes. Furthermore, ACE2 protein expression was significantly downregulated in hydrogen peroxide-stimulated cardiomyocyte lysates with an expected electrophoretic mobility of ~ 110 kDa (Fig. 6A). However, ACE2 mRNA expression level was upregulated in hydrogen peroxide-stimulated cardiomyocyte (Fig. 6B). To further evaluate this uncoupling phenomenon of ACE2, apical supernatants were collected from treated cardiomyocytes. Medium was concentrated by centrifugation in ultra-centrifugal filters (Millipore, cat # UFC800308) to collect free protein from the supernatant. ACE2 secreted protein, a fragments with a electrophoretic mobility of ~ 90 kDa, was observed in the concentrated supernatants, suggesting the release of an ACE2 fragment from cells. Western blotting showed an increase in ACE2 secreted protein fragments in injured cardiomyocyte (Fig. 6C). Although ACE2 mRNA level was compensatory increased in injured cardiomyocytes, ADAM17 did not affect ACE2 mRNA expression in these cells (Fig. 6D). We verified the direct association between ACE2 and ADAM17 further. Co-immunoprecipitation experiments showed that ADAM17 co-immunoprecipitated with ACE2 (Fig. 6E). Hydrolytic cleavage of ACE2 was significantly inhibited by ADAM17 inhibition, as evidenced by increased expression of ACE2 protein with an electrophoretic mobility of approximately 110 kDa in cell lysates, while the level of ~ 90 kDa fragment in the supernatant was decreased (Fig. 6F–H). Meanwhile, the activity of ACE2 also changed accordingly. The activity of ACE2 in the lysate increased upon inhibition of ADAM17 (Fig. 6I) while was decreased in the supernatant (Fig. 6J). The above results suggest that ADAM17 may regulate the functional state of MI cardiomyocytes by regulating the ectodomain shedding of ACE2.

### ADAM17 activation is dependent on P38MAPK

**Fig. 7:**
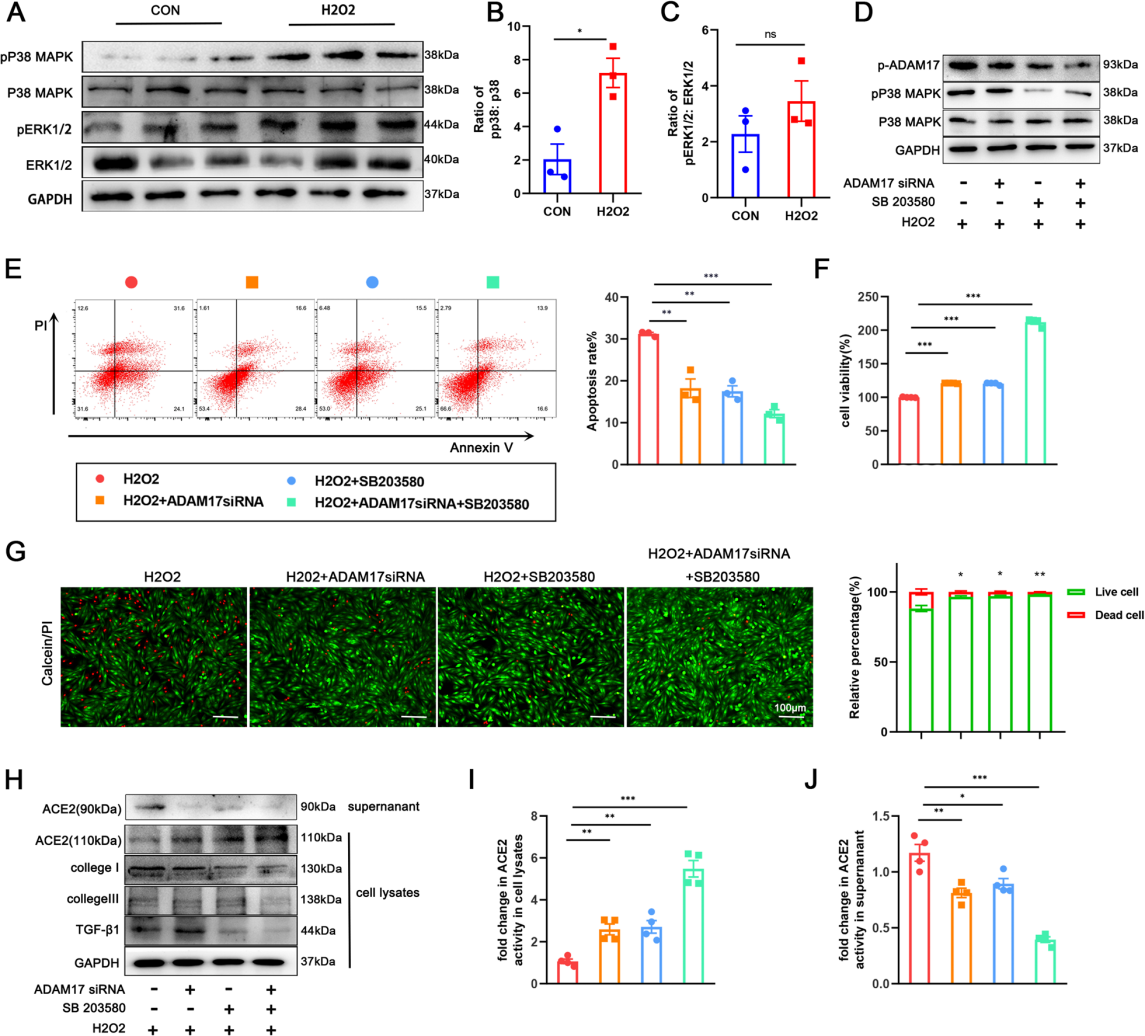
Activated P38MAPK induced phosphorylation of ADAM17 in injured cardiomyocyte. **A**–**C** Representative western blot (A) and quantitative results depicting the phosphorylation vs. total protein levels of P38 (B) and ERK1/2 (C) in H9C2 cardiomyocytes treated with 600 μM hydrogen peroxide for 12 h (n = 3). **D** Phosphorylated ADAM17 (p-ADAM17) was assessed by western blot with anti-phospho-ADAM17 (pThr^735^) antibody after transfection with ADAM17 siRNA or P38MAPK inhibitor SB203580 as indicated (n = 3). **E** Flow cytometric analysis of annexin V/PI double stained cardiomyocyte after treatment as indicated (right). Histogram indicating the percentage of later apoptosis cells in 4 groups (left) (n = 3). **F** The cell viability was detected by CCK8 assay (n = 4). **G** The live (green) and dead (red) cells were observed by calcein-AM/PI double staining kit after treatment as indicated (right). Histogram showing percentage of viable cells(left) (n = 3). **H** Protein levels of ACE2 (110 kDa), collagen I, collagen III TGF-β1 in cardiomyocytes and released ACE2 protein (90 kDa) in the supernatant were detected by western blotting(n = 3). **I**, **J** ACE2 activity in cardiomyocyte lysates (I) and cardiomyocyte supernatant (J) was detected by the ACE2 Activity Fluorometric Assay(n = 4). Data shown as mean ± SEM. Derived by two-sample t-test, *P < 0.05; **P < 0.01, ***P < 0.001

The transmembrane domain of ADAM17 was necessary for the hydrolase activity. It’s kown that the threonine phosphorylation of the cytoplasmic tail of ADAM17 by ERK1/2 or P38-MAPK increase the ability of ADAM17 to cleave substrates. First, the potential phosphorylation sites on the ADAM17 sequence were screened using the SCANSITE database (http://scansite.mit.edu), and the results suggested that P38MAPK had a strong correlation with the Thr735 phosphorylation of ADAM17 (p-ADAM17) (Additional file 1: Figure S4A). Therefore, we tested for changes in ADAM17 phosphorylation in stimulated cells using anti-phospho-ADAM17 (pThr735) antibodies and were surprised to find increased ADAM17 phosphorylation compared to controls (Additional file 1: Figure S4B). We examined the expression and activity of ERK1/2 and P38MAPK to gain further insight into the parameters that support ADAM17 sheddase activity. Western blot showed that phosphorylated P38 (pP38) but not phosphorylated ERK1/2 (pERK1/2) were significantly upregulated in injured cardiomyocytes as compared with controls group (Fig. 7A–C). These data illustrate that ADAM17 phosphorylation may via P38 MAP kinase, but not ERK1/2 MAPK pathway.

Next, we regulated the expression of ADAM17 and P38MAPK, respectively. Either the P38MAPK inhibitor SB203580 (10 μM) or ADAM17 siRNA both eliminated the increased p-ADAM17 after hydrogen peroxide-stimulation (Fig. 7D). When these two inhibitors were used together, the expression of p-ADAM17 was more significantly reduced. Similar to the above findings, the apoptosis of injured cardiomyocytes was decreased (Fig. 7E); cell viability was increased (Fig. 7F); the quantity of viable cardiomyocytes was increased (Fig. 7G); and the expression of myocardial remodeling-related proteins was decreased (Fig. 7H) after blocking ADAM17 and P38MAPK, respectively. Combined suppression of ADAM17 and P38MAPK greatly reduced cardiomyocyte apoptosis and remodeling, indicating that function of ADAM17 may be partially dependent on P38MAPK. The expression of ACE2 in cell lysates and supernatants was then investigated. As expected, suppression of ADAM17 and P38MAPK greatly raised total ACE2 expression in cell lysate and restored ACE2 activity, whereas expression of ACE2 cleavage protein fragments in serum was reduced and ACE2 activity was decreased (Fig. 7I, J). This is significant because it raises the notion that P38MAPK activation is required to trigger ADAM17 phosphorylation for the cleavage of ACE2.

In order to exclude the crosstalk with other p38 MAPK specific substrates, three P38 MAPK downstream substrates, MAPKAP kinase 2(MK2) [40–42], STAT3 [43] and activating transcription factor 2 (ATF2) [44], related to apoptosis and fibrosis were identified. It has been confirmed that phosph-MK2 and phospho-ATF2 is associated with P38 MAPK activation. Thus, we detected phosph-MK2, STAT3 and phospho-ATF2 protein levels in the injured cardiomyocytes. Our results showed that phosph-MK2 was up-regulated, but the expressions of STAT3 and phospho-ATF2 were not significantly affected (Additional file 1: Figure S4C). In general, changes in phosph-MK2 expression were smaller than those of ADAM17, but their cross-talk with P38 MAPK could not be excluded. Previous studies have demonstrated that the p38MAPK/MK2 signaling pathway can lead to redox stress, cell death, and ischemia/reperfusion injury, however, the correlation between MK2 and ADAM17 has not been reported. Thus, the role and mechanism of MK2 by P38MAPK in designing the authors for HF after MI needs further investigation.

### ADAM17 aggravates post-MI HF progression in mice by mediated shedding of ACE2

**Fig. 8:**
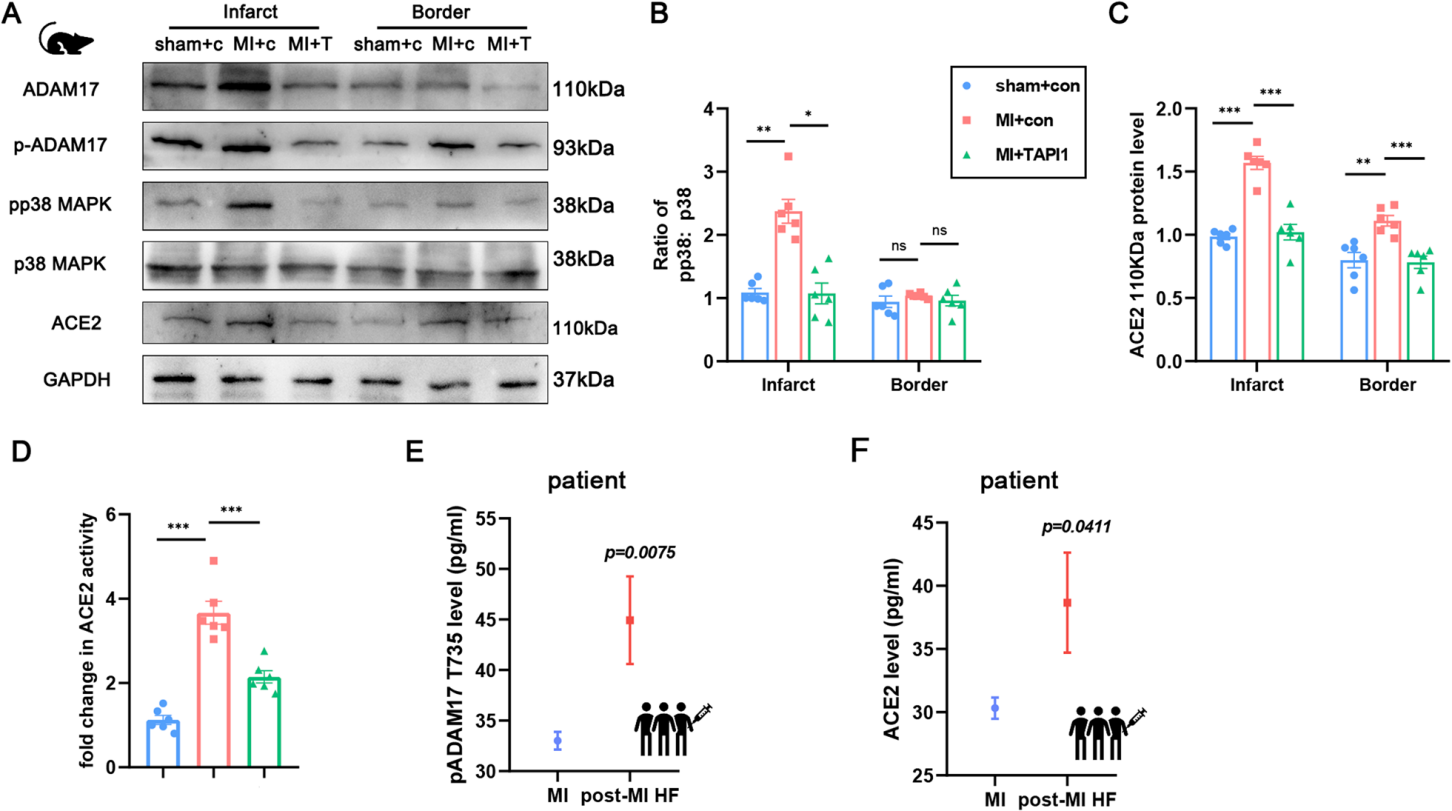
ADAM17 deletion reduces ACE2 shedding in post-MI mice. Knockout of ADAM17 in the myocardium of MI mice using TAPI-1, the same as before. **A** Western blot analysis of the expression of ADAM17, p-ADAM17, pP38MAPK, total P38MAPK, ACE2 protein in different position myocardium of 30 days post-MI mice. **B** Quantification of pP38MAPK levels in different position of myocardium. **C** Quantification of ACE2 levels in cardiomyocytes. **D** Detection of ACE2 activity in cardiomyocyte lysates by ACE2 activity fluorometric assay. **E** Detection of pADAM17 Thr^735^ expression in peripheral blood of patients with or without HF after MI. **F** Detection of ACE2 expression in peripheral blood of patients with or without HF after MI. N = 6 biological replicates. Data shown as mean ± SEM. Derived by two-sample t-test, *P < 0.05; **P < 0.01, ***P < 0.001

**Fig. 9:**
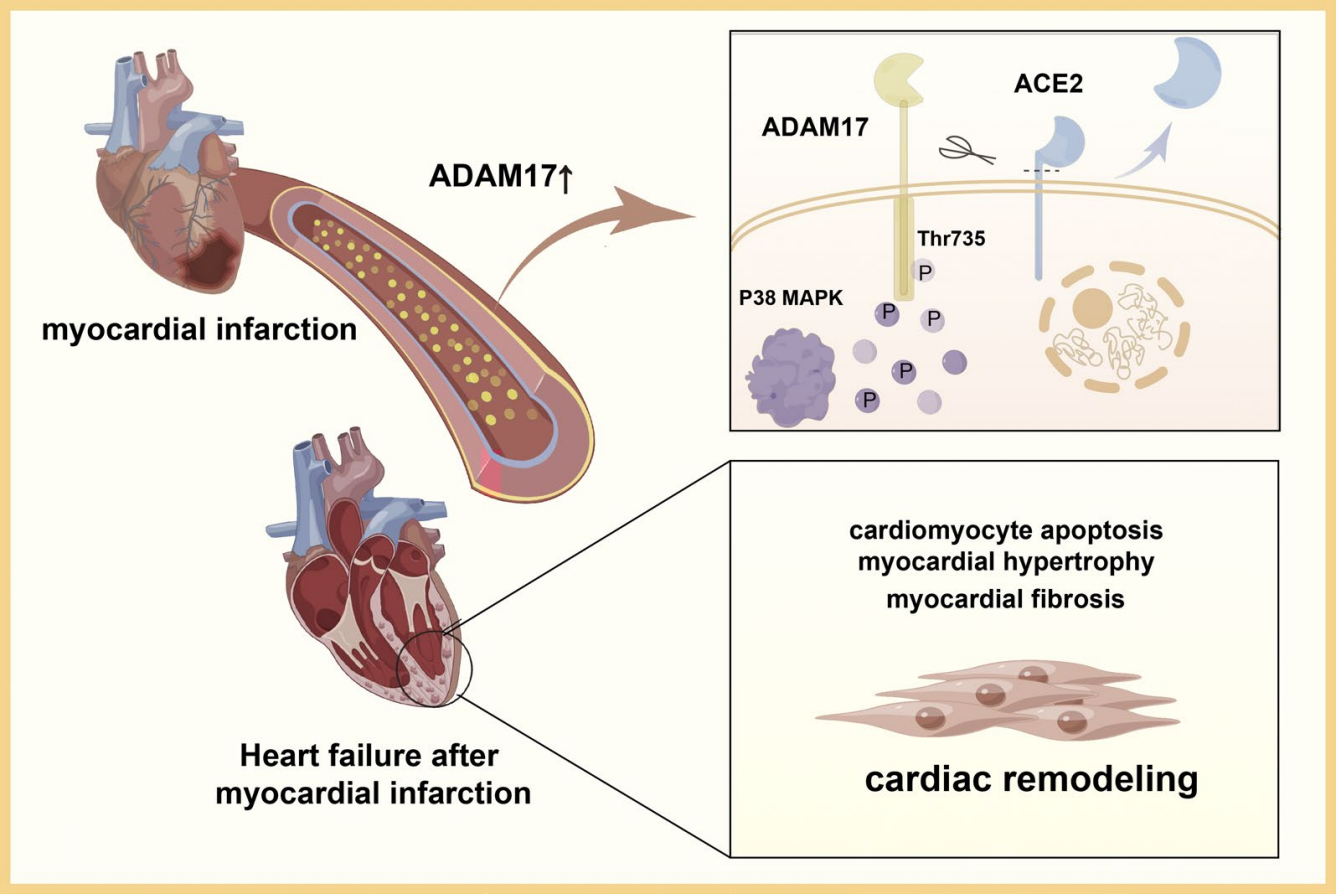
Graphical summary. Our findings highlight the potential translational value of ADAM17 as an early warning biomarker of post-MI HF. ADAM17 activated by P38MAPK promotes the hydrolytic shedding of the cardiomyocyte transmembrane protein ACE2 and mediated the development of cardiac remodeling

To further verify the mechanism of ADAM17, an ADAM17 knockout MI mouse model was constructed by using TAPI-1 in the same way as before (Fig. 3A). After 30 days, the heart tissue was collected for western blot analysis. The expression of ADAM17 and p-ADAM17 were elevated in both of the infarct and remote myocardium of MI mice, while in ADAM17-blocked MI mice were inhibited (Fig. 8A). And the expression of ADAM17 was generally higher at infarcted sites than in remote sites. P38MAPK activation, manifested as a higher ratio of pP38 vs. P38, was also found after MI (Fig. 8B). However, after inhibition of ADAM17 expression, P38MAPK activation was significantly reduced in the infarcted region but not in the distal myocardium, suggesting that P38MAPK activation was mainly concentrated in the region of cardiac injury. As for ACE2, myocardial ACE2 expression was significantly increased after MI and decreased in ADAM17 blocked MI mice (Fig. 8C). The activity of ACE2 was also increased after MI and decreased in ADAM17 blocked MI mice (Fig. 8D). Additionally, we also found that the expressions of pADAM17 and soluble ACE2 were significantly increased in the peripheral blood of patients with HF within 1 year after MI, which further confirmed this mechanism (Fig. 8E, F). This change occurred within 24 h after MI, suggesting that ADAM17 maybe have been involved earlier, but larger data are still needed. Therefore, our result suggested that ACE2 can be released from the cardiomyocyte membrane through ADAM17-mediated proteolytic cleavage, resulting in loss of the protective function of ACE2 and aggravating cardiomyocyte remodeling (Fig. 9).

## Discussion

Heart failure after an acute myocardial infarction (AMI) is a significant cause of morbidity and mortality worldwide [45]. Our findings reveal that ADAM17-driven the shedding of ACE2 is an obligate requirement for HF after MI. We highlight a novel and important mechanism for ADAM17 to cause post-MI HF, which will hopefully be a new potential target for early prediction or intervention of post-MI HF. P38MAPK triggers ADAM17 activation, leading to shedding of the transmembrane protein ACE2 and exacerbating cardiomyocyte necrosis and remodeling in infarcted cardiomyocytes. Reducing ADAM17 activity enhanced cardiac function and long-term prognosis in MI mice by preventing adverse cardiac remodeling and inhibiting myocardial cell apoptosis.

ADAM17 is a crucial protease for post-transcriptional modification [46]. According to earlier research, ADAM17 exhibits dynamic changes in cardiac remodeling after MI [20, 47]. Cardiomyocyte-specific ADAM17 knockdown mice promotes myocardial infarction recovery by accelerating angiogenesis [47]. ADAM17 has previously been shown to regulates pressure overload-induced myocardial hypertrophy and dysfunction through proteolytic processing of integrin β1 [48]. Additionally, ADAM17 knockdown in cardiomyocytes was sufficient to suppress VEGFR2 induction and activation, impairing angiogenesis in the infarct and peri-infarct myocardium [49]. ADADM17 can also be regulated by cardiac MiR-26a-5p, to reduce the apoptosis of myocardial cells and the expression of inflammatory factors in acute myocardial infarction [50]. These data suggest a potential benefit of ADAM17 inhibition on MI prognosis. Here our data suggest that ADAM17, which drives the shedding of ACE2, may participate the occurrence of HF after MI. The follow-up data support further investigation of this receptor as a potential novel target for modifying prognosis in post-MI HF patients. ADAM17 represents a novel biomarker, which offers prognostic information in patients with post-MI HF that is independent of clinical predictors. The present report extends prior work demonstrating an association between ADAM17 and mortality among patients with post-MI HF. A high level of ADAM17 is a hallmark of post-MI HF associated with poor prognosis being an independent predictor of mortality.

The extracellular structural domain of ADAM17 sheds to mediate cardiovascular disease. ADAM17 cleaves a wide range of substrates, including growth factors, cytokines, receptors, and adhesion molecules, which then start or modify intracellular signaling [51]. In vitro mechanism studies revealed that ADAM17 mediates ACE2 extracellular shedding and inhibits the cardioprotective function of ACE2. And this process depends on the phosphorylation of P38MAPK. Additionally, ADAM17 knockout MI mice also showed the comparable changes in ACE2 and P38MAPK, but more specific target regulation in vivo is needed in the future.

The functional state of ADAM17 is regulated by multiple protein modifications. Certain regulators modulate ADAM17 activity via stabilization. FERM structural domain-containing protein 8 (FRMD8) has been reported to stabilize ADAM17 at the cell surface and support ADAM17-mediated ligand shedding [52]. P38MAPK has also been reported to activate ADAM17 and EGFR signaling [53].

Here, we confirmed that P38MAPK interacts with the cytoplasmic domain of ADAM17 and phosphorylates on Thr^735^, which is required for ADAM17-mediated ectodomain shedding. After inhibiting P38MAPK activity, the expression of ADAM17 was significantly decreased and the remodeling of MI cardiomyocytes was alleviated, indicating that P38MAPK could regulate ADAM17 and participate in the pathogenic process of MI cardiomyocytes. Moreover, co-inhibition of ADAM17 and P38MAPK increased the activity of injured cardiomyocytes more than that of P38MAPK alone, with more significant changes in downstream ACE2 expression and activity. These findings suggest that the function of ADAM17 may be partially dependent on P38MAPK. It is unclear whether there are other influencing factors that need to be investigated further.

ACE2, a type I transmembrane protein with a catalytically active ectodomain exposed to the circulation, is a known marker of poor prognosis in patients with cardiovascular disease [54, 55]. In this study, we demonstrate the soluble form of ACE2 can be released from the membrane via ADAM17 mediated proteolytic cleavage, resulting in loss of ACE2 protection against tissue RAS and increased plasma ACE2 activity. Although the ACE2 mRNA level was markedly upregulated in injured myocardial cells probably due to a compensatory mechanism, the ACE2 protein level were not increase in hydrogen peroxide-stimulated cardiomyocyte compared with control. This uncoupling phenomenon between ACE2 mRNA and protein expression levels is supportive of the post-translational regulation of ACE2 due to the proteolytic shedding by ADAM17, whereas the deficiency of ADAM17 resulted in preserved ACE2 on the cardiomyocyte surface. This conclusion was confirmed by the absence of significant differences in ACE2 mRNA expression after further inhibition of ADAM17 expression, which reduced ACE2 expression and activity in cardiomyocytes in vitro and in vivo after ADAM17 inhibition, reduced myocardial remodeling after MI, and improved prognosis. Notably, ACE2 has garnered much attention in view of the current COVID-19 pandemic as the cellular receptor for severe acute respiratory syndrome coronavirus 2 (SARS-CoV-2) [56]. Myocardial injury is a frequent side effect in COVID-19 patients [57]. A cohort researches indicated that 13% of 538 patients with new infected patients experienced cardiovascular-related symptoms 97 days after discharge [58]. Another cohort research by Puntmann VO et al. revealed that 78% of 100 patients developed myocardial involvement 71 days following the onset of new coronary symptoms [59]. Chronic hypoxia and increased pulmonary artery pressure and ventricular strain may further increase the incidence of myocardial injury in patients with COVID-19 [60, 61]. The high expression of ACE2 receptors in the heart provides a direct infection route for SARS-CoV-2, which may be one of the reasons for myocardial injury in patients with COVID-19 [62]. However, the role of Ace2 is a double-edged sword. On the one hand, it is a pathway for viral entry into cells, but at the same time, it can also shield cardiomyocytes from damage. Therefore, the role of ACE2 in the treatment of post-COVID-19 myocardial injury remains controversial. The critical role of ADAM17-ACE2 regulatory pathway in hypoxic myocardium revealed in this study has a certain guider for the management and drug development of patients with heart injury aggravated by COVID-19.

Taken together, our findings highlight the potential translational value of ADAM17 inhibition post-MI HF. The detection of peripheral blood in clinical patients suggests a new potential target for early prediction or intervention of post-MI HF. Cardiomyocytes deletion of ADAM17 inhibit ACE2 shedding, limit ventricular remodeling, alleviate cardiomyocyte death, restricts scar expansion, and preserve cardiac function post-MI. Thus, these observations suggest that selective inhibition of ADAM17 may be a therapeutic strategy to limit MI-induced cardiac remodeling and HF.

Our study has certain limitations. First, the pathophysiological changes of post-MI HF are complex and dynamic. ADAM17, as a key shear enzyme to promote the hydrolysis of ACE2, regulates myocardial remodeling, but the existence of other molecular pathways remains to be further investigated. And more specific target regulation in vivo is needed in the future. Furthermore, we confirmed much higher levels of ADAM17 expression in early peripheral blood of patients with heart failure after MI, suggesting an early warning role for ADAM17, but larger data are still needed.

## Supplementary Information

**Additional file 1**.** Figure S1**: Expression of ADAM17 in rat primary myocardium and fibroblasts. (A–B) Rat primary cardiomyocytes were exposed to the indicated concentrations (A) or time (B) of H2O2 and RT-qPCR was used to measure the expression of ADAM17. (C–D) Rat primary fibroblasts were exposed to the indicated concentrations (C)or time (D) of H2O2 and RT-qPCR was used to measure the expression of ADAM17. N = 3 biological replicates. Data shown as mean ± SEM. Derived by two-sample t-test, *P < 0.05; **P < 0.01, ***P < 0.001.** Figure S2**: Construction of siRNA against ADAM17 expression in H9c2 cells. H9C2 cells were transfected with a ADAM17 siRNA or siNC for 6h before H2O2 treatment. (A) RT-qPCR for ADAM17 mRNA in H9C2 cells treated with siRNA as described above. (B) Western blot analysis of the expression of ADAM17 in H9C2 cells treated with siRNA as described above. N=3 biological replicates. Data shown as mean ± SEM. Derived by two-sample t-test, *P < 0.05; **P < 0.01.** Figure S3**: ADAM17 interacts with ACE2. (A) ADAM17 (PDB: 2FV9, green) docks to ACE2 (PDB: 6M17, purple). (B) Hydrophobic and (C) hydrogen bonds at the interaction site. Pymol 2.3.0 Interaction mode for analyzing docking results.** Figure S4**: ADADM17 is phosphorylated by P38MAPK. (A) The scansite database (http://scansite.mit.edu) suggests an interaction site between the ADAM17 threonine 735 site and P38MAPK. (B) Western blot analysis of the expression of p-ADAM17 in H9C2 cells treated with 600μM H2O2 for 12h. (C) Western blot analysis of the expression of p-MK2, STAT2, p-ATF2 in H9C2 cells treated with 600μM H2O2 for 12h. Data shown as mean ± SEM. N=3 biological replicates. Derived by two-sample t-test, *P < 0.05.** Table S1**: Baseline characteristics of MI patients with and without new HF onset at one-year follow-up.** Table S2**: Primers used in study.** Table S3**: Troponin I expression in serum of MI mice.

## Data Availability

Not applicable.
