# Supplementary material for: P38 MAPK activated ADAM17 mediates ACE2 shedding and promotes cardiac remodeling and heart failure after myocardial infarction
Source: Cell Commun Signal. 2023 Apr 12;21:73. doi: 10.1186/s12964-023-01087-3 (PMC10091339; doi:10.1186/s12964-023-01087-3)
Supplement: Supplementary file 2 — Additional file 1. Figure S1: Expression of ADAM17 in rat primary myocardium and fibroblasts. (A–B) Rat primary cardiomyocytes were exposed to the indicated concentrations (A) or time (B) of H2O2 and RT-qPCR was used to measure the expression of ADAM17. (C–D) Rat primary fibroblasts were exposed to the indicated concentrations (C)or time (D) of H2O2 and RT-qPCR was used to measure the expression of ADAM17. N = 3 biological replicates. Data shown as mean ± SEM. Derived by two-sample t-test, *P < 0.05; **P < 0.01, ***P < 0.001. Figure S2: Construction of siRNA against ADAM17 expression in H9c2 cells. H9C2 cells were transfected with a ADAM17 siRNA or siNC for 6h before H2O2 treatment. (A) RT-qPCR for ADAM17 mRNA in H9C2 cells treated with siRNA as described above. (B) Western blot analysis of the expression of ADAM17 in H9C2 cells treated with siRNA as described above. N=3 biological replicates. Data shown as mean ± SEM. Derived by two-sample t-test, *P < 0.05; **P < 0.01. Figure S3: ADAM17 interacts with ACE2. (A) ADAM17 (PDB: 2FV9, green) docks to ACE2 (PDB: 6M17, purple). (B) Hydrophobic and (C) hydrogen bonds at the interaction site. Pymol 2.3.0 Interaction mode for analyzing docking results. Figure S4: ADADM17 is phosphorylated by P38MAPK. (A) The scansite database (http://scansite.mit.edu) suggests an interaction site between the ADAM17 threonine 735 site and P38MAPK. (B) Western blot analysis of the expression of p-ADAM17 in H9C2 cells treated with 600μM H2O2 for 12h. (C) Western blot analysis of the expression of p-MK2, STAT2, p-ATF2 in H9C2 cells treated with 600μM H2O2 for 12h. Data shown as mean ± SEM. N=3 biological replicates. Derived by two-sample t-test, *P < 0.05. Table S1: Baseline characteristics of MI patients with and without new HF onset at one-year follow-up. Table S2: Primers used in study. Table S3: Troponin I expression in serum of MI mice. [file 12964_2023_1087_MOESM2_ESM.docx]

**supplementary materials**

**
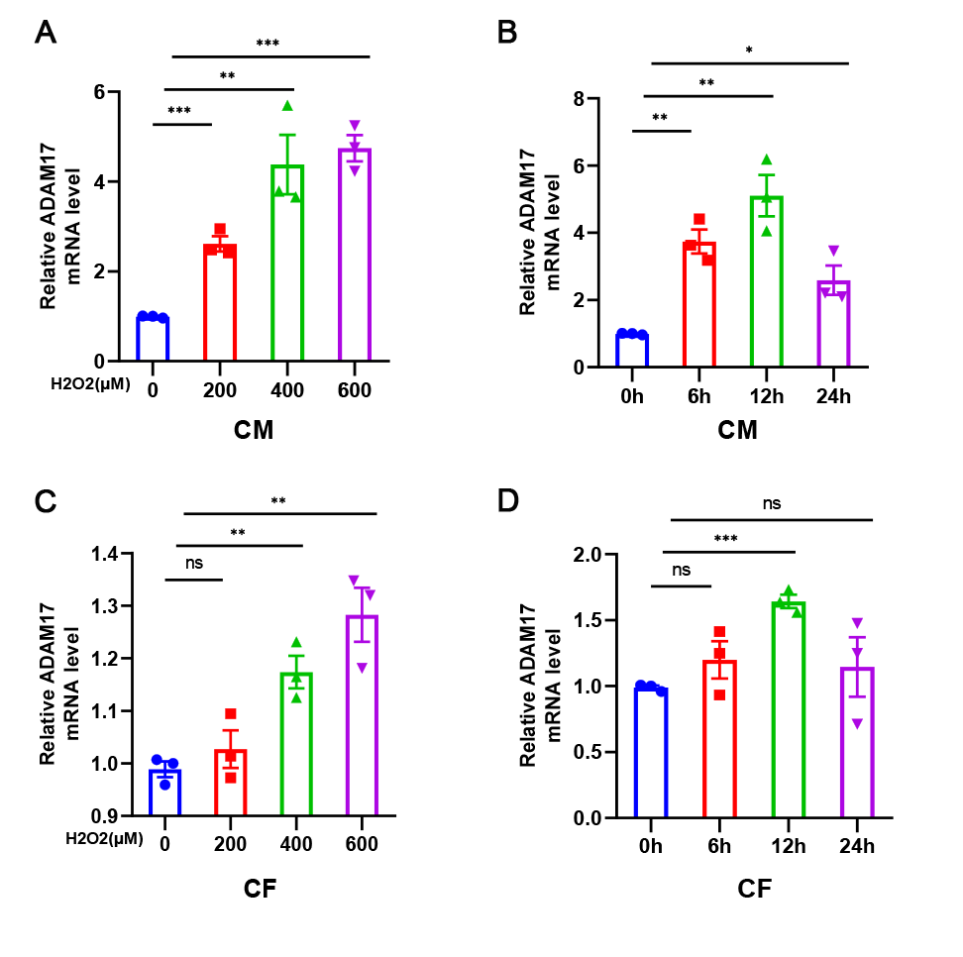
**

**Supplementary Figure 1** Expression of ADAM17 in rat primary myocardium and fibroblasts. **(A-B)** Rat primary cardiomyocytes were exposed to the indicated concentrations (A)or time (B) of H2O2 and RT-qPCR was used to measure the expression of ADAM17. **(C-D)** Rat primary fibroblasts were exposed to the indicated concentrations (C)or time (D) of H2O2 and RT-qPCR was used to measure the expression of ADAM17. N=3 biological replicates. Data shown as mean ± SEM. Derived by two-sample t-test, *P <0.05; **P<0.01, ***P<0.001.


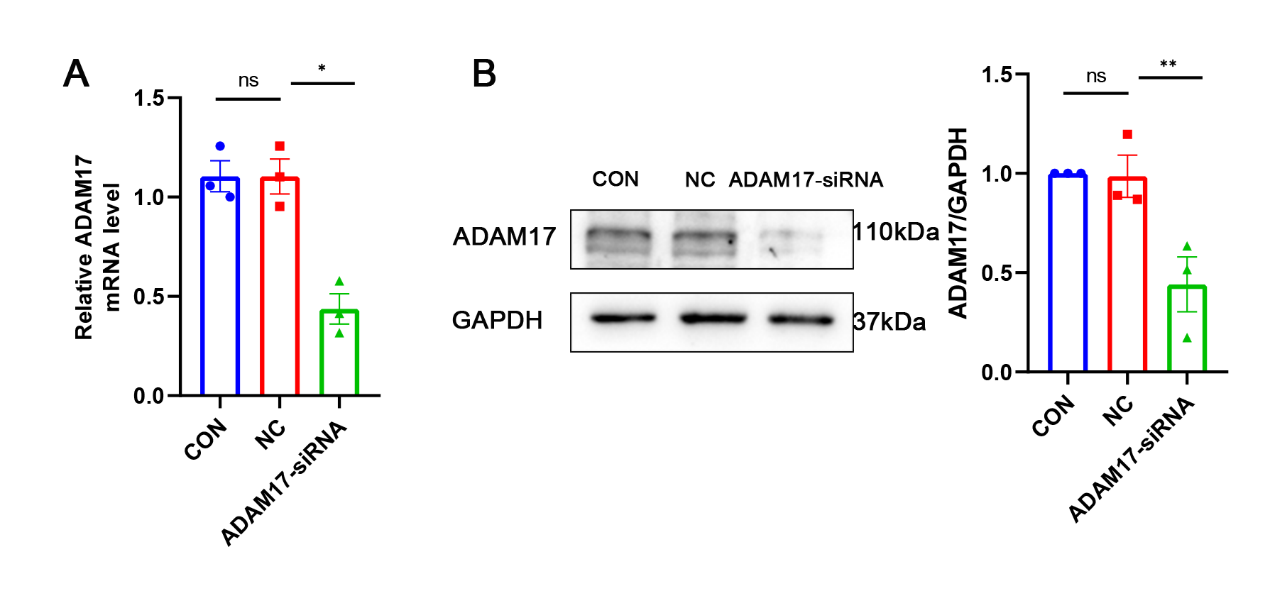


**Supplementary Figure 2** Construction of siRNA against ADAM17 expression in H9c2 cells. H9C2 cells were transfected with a ADAM17 siRNA or siNC for 6h before H2O2 treatment. **(A)** RT-qPCR for ADAM17 mRNA in H9C2 cells treated with siRNA as described above. **(B)** Western blot analysis of the expression of ADAM17 in H9C2 cells treated with siRNA as described above. N=3 biological replicates. Data shown as mean ± SEM. Derived by two-sample *t*-test, **P* <0.05; ***P*<0.01.


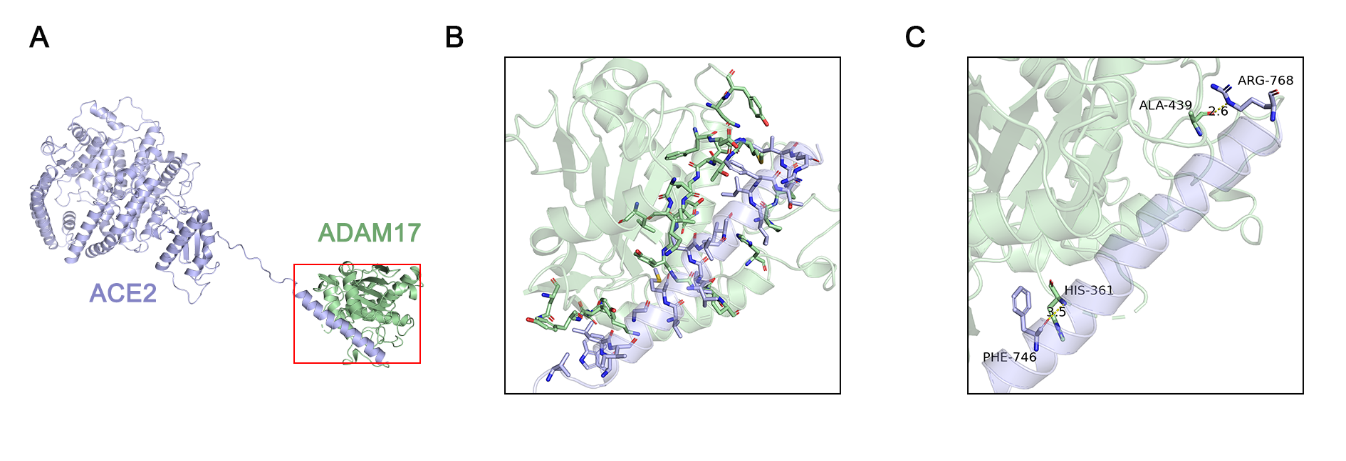


**Supplementary Figure 3** ADAM17 interacts with ACE2. **(A)** ADAM17 (PDB: 2FV9, green) docks to ACE2 (PDB: 6M17, purple). **(B)** Hydrophobic and **(C)** hydrogen bonds at the interaction site. Pymol 2.3.0 Interaction mode for analyzing docking results.


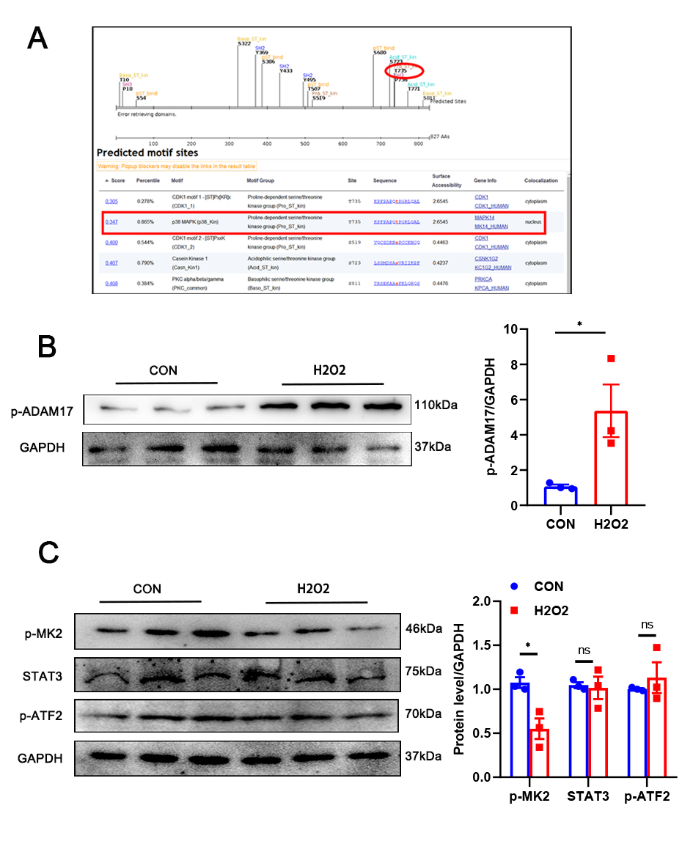


**Supplementary Figure 4** ADADM17 is phosphorylated by P38MAPK. **(A)** The scansite database (http://scansite.mit.edu) suggests an interaction site between the ADAM17 threonine 735 site and P38MAPK. **(B)** Western blot analysis of the expression of p-ADAM17 in H9C2 cells treated with 600μM H2O2 for 12h. **(C)** Western blot analysis of the expression of p-MK2, STAT2, p-ATF2 in H9C2 cells treated with 600μM H2O2 for 12h. Data shown as mean ± SEM. N=3 biological replicates. Derived by two-sample *t*-test, **P* <0.05.

**Supplementary Table1.** Baseline characteristics of MI patients with and without new HF onset at one-year follow-up.

| Characteristic | Overall | Non-HF | HF | *P*-value |
| --- | --- | --- | --- | --- |
|  | N =152 | N =76 | N =76 |  |
|  | *Baseline characteristics* | | |  |
| Age, years | 65[58,71] | 64[56,68] | 67[60,74] | 0.007 |
| Gender |  |  |  |  |
| Male, no. (%) | 86(56.579) | 45(59.211) | 41(53.947) | 0.513 |
| Female, no. (%) | 66(43.421) | 31(40.789) | 35(46.053) |  |
| Hypertension, no. (%) | 84(55.263) | 36(47.368) | 48(63.158) | 0.05 |
| Diabetes, no. (%) | 47(30.921) | 20(26.316) | 27(35.526) | 0.219 |
| Cerebrovascular disease, no. (%) | 35(23.026) | 14(18.421) | 21(27.632) | 0.177 |
| Smoking(current+ex) | 73(48.026) | 36(47.368) | 37(48.684) | 0.871 |
| Drinking(current+ex) | 21(13.816) | 9(11.842) | 12(15.789) | 0.481 |
|  |  | *Laboratory covariates* |  |  |
| cTnI,μg/L | 4.059[0.866,18.033] | 2.791[0.906,18.033] | 5.565[0.698,17.842] | 0.64 |
| hs-CRP, mg/L | 9.007±5.643 | 7.233±5.480 | 11.053±5.112 | <0.001 |
| Hemoglobin, g/L | 131.928±23.268 | 135.145±22.305 | 128.711±23.762 | 0.089 |
| eGFR, mL/min/1.73 m^2^ | 75.106[56.783,90.520] | 84.910[71.285,94.727] | 67.508[44.773,81.873] | <0.001 |
| Scr, μmol/L | 85[72,105] | 80[70,94] | 97[73,130] | 0.001 |
| Urea, mmol/L | 6.19[4.98,8.48] | 5.5[4.45,7.09] | 7.01[5.5,9.84] | <0.001 |
| Glucose, mmol/L | 8.419±3.95 | 7.667±3.605 | 9.192±4.136 | 0.018 |
| Sodium, mmol/L | 138[135.8,140.1] | 138[136,140] | 137.2[135.5,140.3] | 0.477 |
| Potassium, mmol/L | 4[3.7,4.3] | 4[3.7,4.3] | 4.1[3.7,4.4] | 0.305 |
| Chloride, mEq/L | 103[101,105] | 104[101,105] | 103[101,104.8] | 0.357 |
| Serum albumin, g/L | 39.7±4.872 | 41.1±4.038 | 38.3±5.219 | <0.001 |
| TG, mmol/L | 1.632±1.102 | 1.693±1.145 | 1.561±1.047 | 0.481 |
| TC, mmol/L | 4.514±1.377 | 4.643±1.452 | 4.367±1.271 | 0.238 |
| HDL, mmol/L | 1.106±0.299 | 1.118±0.299 | 1.093±0.298 | 0.631 |
| LDL, mmol/L | 2.745±0.857 | 2.749±0.814 | 2.74±0.904 | 0.949 |
|  |  | *Treatment* |  |  |
| PCI, no. (%) | 101(66.447) | 53(69.737) | 48(63.158) | 0.39 |
| Statins, no. (%) | 127(83.553) | 68(89.474) | 59(77.632) | 0.049 |
| ACEI/ARB, no. (%) | 76(50) | 37(48.684) | 39(51.316) | 0.746 |
| Beta-blocker, no. (%) | 82(53.947) | 40(52.632) | 42(55.263) | 0.745 |
| Spironolactone, no. (%) | 53(34.868) | 17(22.368) | 36(47.368) | 0.001 |
| Diuretic, no. (%) | 29(19.079) | 10(13.158) | 19(25) | 0.063 |
| Calcium antagonist, no. (%) | 10(6.579) | 4(5.263) | 6(7.895) | 0.513 |
| Nitrates, no. (%) | 65(42.763) | 32(42.105) | 33(43.421) | 0.87 |
| OHAs, no. (%) | 11(7.237) | 7(9.211) | 4(5.263) | 0.348 |
| Insulin, no. (%) | 21(13.816) | 8(10.526) | 13(17.105) | 0.24 |

Continuous variables were expressed as mean ± standard deviation or median [IQR], and Student's *t-* test or Mann-Whitney U was used for comparison between groups. Categorical variables were expressed as frequency and percentage (%), and Chi-square test was used for comparison between groups.

**Supplementary Table 2.** Primers used in study

| PCR Primers (5' to 3') |  |  |
| --- | --- | --- |
| ADAM17-RAT | Forward | CAGCAGGTGTCGTTGTTCAGGTAC |
|  | Reverse | CAAGGTGTGCGGCAACTCCAG |
| ACE2-RAT | Forward | AAGCCACCTTACGAGCCTCCTG |
|  | Reverse | ACAATGCCAACCACTACCGTTCC |
| Collagen Ⅰ-RAT | Forward | GTACATCAGCCCAAACCCCA |
|  | Reverse | GGGACTTCTTGAGGTTGCCA |
| Collagen Ⅲ-RAT | Forward | TCTGGCGGCTTTTCACCATA |
|  | Reverse | GCATCCATCTTGCAGCCTTG |
| TGF-β1-RAT | Forward | CTGAACCAAGGAGACGGAATAC |
|  | Reverse | GTTTGGGACTGATCCCATTGA |
| MyHC-RAT | Forward | CCAGAACACCAGCCTCATCAACC |
|  | Reverse | CACCGCCTCCTCCACCTCTG |
| ADAM17-MOUSE | Forward | CGAGTGAAGAGACGAGCTGAACC |
|  | Reverse | CTGTCCCGAGTGCCTGAAATGTAG |
| ANP-MOUSE | Forward | GTGCGGTGTCCAACACAGAT |
|  | Reverse | TCCAATCCTGTCAATCCTACCC |
| BNP-MOUSE | Forward | GAGGTCACTCCTATCCTCTGG |
|  | Reverse | GCCATTTCCTCCGACTTTTCTC |
| MyHC-MOUSE | Forward | CCTGCGGAAGTCTGAGAAGG |
|  | Reverse | CTCGGGACACGATCTTGGC |

**Supplementary Table 3.** **Troponin I expression in serum of MI mice**

| MI mice | overall |
| --- | --- |
| preoperative, mean ± sd | 9.7611 ± 2.8549 |
| Postoperative,12h, mean ± sd | 27.601 ± 5.4285 |

Data are shown as mean ± standard deviation.
